# Joint Beamforming, Power Allocation, and Splitting Control for SWIPT-Enabled IoT Networks with Deep Reinforcement Learning and Game Theory

**DOI:** 10.3390/s22062328

**Published:** 2022-03-17

**Authors:** JainShing Liu, Chun-Hung Richard Lin, Yu-Chen Hu, Praveen Kumar Donta

**Affiliations:** 1 Department of Computer Science and Information Engineering, Providence University, Taichung 43301, Taiwan; chhliu@pu.edu.tw; 2 Department of Computer Science and Engineering, National Sun Yat-sen University, Kaohsiung 80424, Taiwan; 3 Department of Computer Science and Information Management, Providence University, Taichung 43301, Taiwan; ychu@pu.edu.tw; 4 Research Unit of Distributed Systems, TU Wien, 1040 Vienna, Austria; pdonta@dsg.tuwien.ac.at

**Keywords:** joint optimization, deep reinforcement learning, game theory, multi-resource allocation, beamforming, power control, energy harvesting, IoT

## Abstract

Future wireless networks promise immense increases on data rate and energy efficiency while overcoming the difficulties of charging the wireless stations or devices in the Internet of Things (IoT) with the capability of simultaneous wireless information and power transfer (SWIPT). For such networks, jointly optimizing beamforming, power control, and energy harvesting to enhance the communication performance from the base stations (BSs) (or access points (APs)) to the mobile nodes (MNs) served would be a real challenge. In this work, we formulate the joint optimization as a mixed integer nonlinear programming (MINLP) problem, which can be also realized as a complex multiple resource allocation (MRA) optimization problem subject to different allocation constraints. By means of deep reinforcement learning to estimate future rewards of actions based on the reported information from the users served by the networks, we introduce single-layer MRA algorithms based on deep Q-learning (DQN) and deep deterministic policy gradient (DDPG), respectively, as the basis for the downlink wireless transmissions. Moreover, by incorporating the capability of data-driven DQN technique and the strength of noncooperative game theory model, we propose a two-layer iterative approach to resolve the NP-hard MRA problem, which can further improve the communication performance in terms of data rate, energy harvesting, and power consumption. For the two-layer approach, we also introduce a pricing strategy for BSs or APs to determine their power costs on the basis of social utility maximization to control the transmit power. Finally, with the simulated environment based on realistic wireless networks, our numerical results show that the two-layer MRA algorithm proposed can achieve up to 2.3 times higher value than the single-layer counterparts which represent the data-driven deep reinforcement learning-based algorithms extended to resolve the problem, in terms of the utilities designed to reflect the trade-off among the performance metrics considered.

## 1. Introduction

The tremendous growth in wireless data transmission would be a result from the introduction of fifth generation of wireless communications (5G) and will continue in the wireless networks beyond 5G (B5G). In particular, the collaboration between 5G enabled Internet of Things (5G-IoT) and wireless sensor networks (WSNs) will extend the connections between the Internet and the real world and widen the scope of IoT services. In such collective networks, by uploading part of or all of the computing tasks to the edge computing, a mobile edge computing (MEC) technique is developed to reduce the enormous data traffic and huge energy consumption brought by a great number of IoT devices and sensors [1,2]. Even given that, realizing 5G or B5G IoT networks is still challenging due to the limited energies for the IoT devices equipped with batteries. To alleviate this problem, simultaneous wireless information and power transfer (SWIPT) are proposed to effectively and conveniently extend the lifetime of IoT devices, and employed in many related works [3,4,5,6,7]. In fact, SWIPT is a key technique in 5G and B5G because power allocation and interference management are still the crucial issues to be addressed in the communication networks [8,9]. In the border ground, the techniques of power control along with beamforming and interference coordination are usually adopted to increase the signal for data transmissions and improve the data rates received by end-users. However, these techniques by default treat the interference as a harmful impact to data transmissions, and ignore its potential to increase the communication capacity. By contrast, SWIPT opens up the potential by harvesting energy from the ambient electromagnetic sources including the interference signals. Consequently, not only would the benefits be obtained in which devices with SWIPT can transfer the interference into a useful resource, but also there is an advantage that can be taken with the signal-to-noise and interference ratio (SINR) to be increased by SWIPT for the residual energy of IoT devices.

In this work for the scenario that multiple BSs or APs can simultaneously transmit data and energy to their mobile nodes (MNs) in edge, we further show that, when the power control and interference management meet SWIPT, an overall system utility reflecting data rate, energy harvesting, and power consumption at the same time can be conduced to lead the system to an optimal trade-off on these performance metrics. Given that, how to allocate the transmit power, select the beamforming vector, and decide the power splitting ratio for the system will be a complex multiple resource allocation (MRA) problem, and can be formulated as a mixed integer nonlinear programming (MINLP) problem or even a non-convex MINLP problem. In general, MINLP problems are NP-hard and no efficient global optimal algorithm is available. Thus, apart from traditional optimization programming programs [10,11,12,13,14,15,16,17], research efforts usually resort to game theory [18,19,20,21], graph theory [22,23], and heuristic algorithms [24,25] to reduce the complexity.

More recently, inspired by the success of deep reinforcement learning (DRL) [26] on the application of computer science in various important fields, using DRL to solve the network problems, such as power control [27,28,29], joint resource allocation [30,31], and energy harvesting [32], becomes one of the main trends in the communication society. Although DRL is a useful tool to resolve these problems, the data-driven approaches that resulted usually treat a given resource optimization problem as a black box to learn its input/output relationship via various DRL techniques, which do not explicitly take the advantages from the model-based counterparts, such as game theory, graph theory, and heuristic algorithms mentioned previously. By noticing this fact, in this work, we first show how to design DRL-based approaches operated in a single layer to (1) jointly solve for power control, beamforming selection, and power splitting decision, and (2) approach the optimal trade-off among the performance metrics without exhaustive search in the action space. Next, we show how to incorporate a data-driven DRL-based technique and a model-driven game-theory-based algorithm to form a two-layer iterative approach to resolve the NP-hard MRA problem. By taking benefits from both data-driven and model-driven methods, the proposed two-layer MRA approach is shown to outperform the single-layer counterparts which rely only on the data-driven DRL-based algorithms.

### 1.1. Related Work

As a related work for LTE, the almost blank subframe (ABS) method was proposed in the standard [33] to resolve the co-channel inter-cell interference problem caused by two LTE base stations interfering with each other. Although ABS works well in fixed beam patterns, it was shown in [34] that ABS would be inefficient due to the dynamic nature of beamforming. Apart from the standard’s solution, particular attention has also been paid to the efforts on resolving different resource allocation (RA) problems. In this work, these efforts would be classified into two categories, namely *model-driven methods* and *data-driven methods*. According to our subjects, the former includes optimization methods and game theory methods while the latter simply denotes machine learning methods. As expected, a lot of previous works would be classified into the former, including graph theory [35,36], optimization decomposition [10,11,13,14,15,17], and dual Lagrangian method [12,16], in addition to game theory.

As a kind of data-driven method in the latter, which requires no model-oriented analysis and design, DRL would play a key role in solving RA problems. For example, the work in [37] proposed an inter-cell interference coordination and cell range expansion technique in heterogeneous networks, wherein dynamic Q-learning-based methods were introduced to improve user throughput. In addition, the previous works [29,38,39] introduced different deep Q-learning-based power control methods to maximize their objectives. Apart from Q-learning, in [40,41], actor–critic reinforcement learning (ACRL) algorithms were developed to reduce energy consumption. Recently, with deep deterministic policy gradient (DDPG), an algorithm was proposed in [32] that can be applicable for continuous states to realize continuous energy management, getting rid of the curse of dimensionality due to discrete action space from Q-learning.

Apart from the above, game-theory-based methods also received a lot of attention. For example, non-cooperative interference-aware RA has been proposed in [19] to improve the resource utilization efficiency of OFDMA networks. In [42], an interference coordination game was introduced, and the Nash equilibrium was found to reduce its computational complexity. Similarly, a joint transmit power and subchannel allocation problem was considered in [20], and a distributed non-cooperative game-based RA algorithm and a linear pricing technique were introduced therein to find the solutions. In addition, a power control problem for self-organizing small cell networks was formulated as a non-cooperative game in [21], which can then be solved by using the distributed energy efficient power control scheme proposed. Recently, by introducing a time-varying interference pricing with SWIPT, the authors in [18] modeled the power allocation problem as a non-cooperative game, and, by minimizing the total interferences experienced, they modeled the subchannel allocation problem as a non-cooperative potential game. Then, they proposed iterative algorithms to obtain the Nash equilibrium points corresponding to these games for the solutions.

More recently, there are different learning-based approaches proposed to resolve various problems in IoT networks. For example, a beamforming design for SWIPT-enabled networks was introduced in [43], where the rate-splitting scheme and the power-splitting energy harvesting receiver are adopted for secure information transfer and energy harvesting, respectively. This work formulates an energy efficiency (EE) maximization problem and properly addresses the beamforming design issue. However, such an issue is not our focus. In [44], an EE maximization problem is considered for the SWIPT enabled heterogeneous networks (HetNets). To resolve this problem, the authors introduced a min-max probability machine and an interactive power allocation/splitting scheme based on convex optimization methods. In the latter, the Lagrange multipliers for the optimization problem involved are obtained by using the subgradient method, which could be time-consuming to converge. Despite the different design aim, our work instead develops a game-based interactive method additionally controlled by a threshold to meet our time constraint. In [45], a sum rate maximization problem was formulated for SWIPT enabled HetNets, which jointly optimizes transmit beamforming vectors and power splitting ratios. With the multi-agent DDPG method for the user equipment (UE) without mobility, this work exhibits a notable performance gain when compared with the fixed beamforming design. When UE is mobile and not in the same location vicinity, the wireless channel is not constant and varies with UE’s location. Taking this into account, the work in [46] resolved the dynamic problem with a multi-agent formulation to learn its optimization policy. Specifically, the authors resorted to the majorization–minimization (MM) technique and Dinkelbach algorithm to find the locally optimal solution using the convex optimization method for solving the power and time allocation problem involved. As a complement to these works, our approach considers single agent-based reinforcement learning to comply with the fact noted in [47] that, when a multi-agent setting is modified by the actions of all agents, the environment becomes non-stationary, and the effectiveness of most reinforcement learning algorithms would not hold in non-stationary environments [48]. In addition, by further collaborating with the game-based iterative algorithms, our approach would reduce the overhead resulting from, e.g., the MM approach to resolve a complex optimization problem such as that in [46].

### 1.2. The Motivations and Characteristics of This Work

In recent years, advances in artificial intelligence are further helped by the neural networks such as generative adversarial networks [49] which use advanced game theory techniques to deep learn information and could converge to the Nash equilibrium of the game involved. In general, these advances can be reflected by the notion that a machine (computer) can learn about the outcomes of the game involved and teaches itself to do better based on the probabilities, strategies, and previous instances of the game and other players under the ground of game theory. By extending the advanced notation to the optimization framework, in this work, we further exhibit the possibility of applying learning-based methods, model-based methods, or both to resolve the joint beamforming, power control, and energy harvesting problem in the SWIPT-enabled wireless networks that can alleviate the hardness of finding an optimal solution with an optimization tool required to be completed in time. In particular, in this scenario, apart from BS *i* serving the user or MN needing to decide its transmit power, beamforming vector, and power splitting ratio, the other BSs j≠i would make their own decisions at the same time, which can affect the user or MN served by BS *i* simultaneously. Here, by leveraging the scenario, we conduct our approach to make a good trade-off between information decoding and energy harvesting, which can be deployed in an actual SWIPT-enabled IoT network as one of the various SWIPT applications surveyed in [50]. Specifically, by using the UE coordinates as that in [51] sent to BS, it can align with the industry specification [33] through the slight modification to reduce the original signal overhead of [33] on the channel state information to be sent by UE with a report to have its length equal to the number of antenna elements at least. As a summary, we list the characteristics of this work as follows:

- We introduce two single-layer algorithms based on the conventional DRL-based models, DQN and DDPG, to solve the joint optimization problem formulated here as a non-convex MINLP problem, and realized as an MRA problem subject to the different allocation constraints.
- We propose further a two-layer iterative approach that can incorporate the capability of data-driven DQN technique and the strength of non-cooperative game theory model to resolve the NP-hard MRA problem.
- For the two-layer approach, we also introduce a pricing strategy to determine the power costs based on the social utility maximization to control the transmit power.
- With the simulated environment based on realistic wireless networks, we show the results that, by means of both learning-based and model-based methods, the two-layer MRA algorithm proposed can outperform the single-layer counterparts introduced which rely only on the data-driven DRL-based models.

The rest of this paper is structured as follows. In Section 2, we introduce the network and channel models for this work. Next, we present the single-layer learning-based approaches in Section 3, followed by the two-layer hybrid approach based on game theory and deep reinforcement learning in Section 4. These approaches are then numerically examined in Section 5 to show their performance differences. Finally, conclusions are drawn in Section 6.

## 2. Network and Channel Models

### 2.1. Network Model

**Figure 1:**
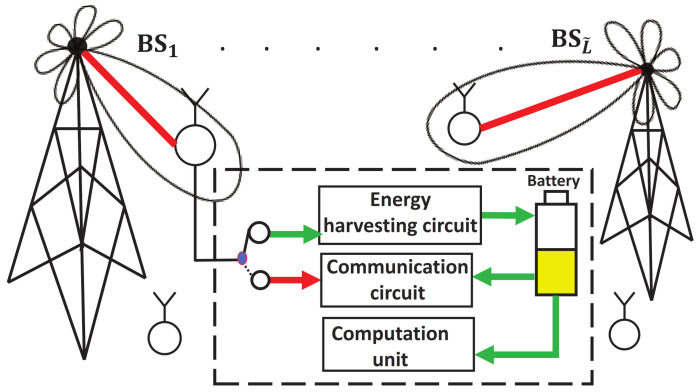
A system model with respect to the joint beamforming, power allocation, and splitting control for SWIPT-enabled IoT networks. In this model, each mobile node has a power split mechanism to split the received signal into two streams, one sent to the energy harvesting circuit for harvesting energy and the other to the communication circuit for decoding information.

As shown in Figure 1, an orthogonal frequency division multiplexing (OFDM) multi-access network with $\tilde{L}$ base stations (BSs) (or access points (APs)) is considered for downlink transmission, in which a serving BS would associate with one mobile node (MN). The distance between two neighbor BSs is *R* and the cell radius (or transmission range) of BS is $\hat{r}>R/2$ to allow overlap. Here, unlike the conventional coordinated multipoint Tx/Rx (CoMP) system applied to the scenario in which a MN could receive data from multiple BSs, we apply the SWIPT technique to the network so that an MN can simultaneously receive not only wireless information but also energy from different BSs. In addition, although mmWave brings many performance benefits as an essential part of 5G, it is also known to have high propagation losses due to higher mmWave frequency bands to be adopted. Thus, analog beamforming for the downlink transmission is considered to alleviate these losses.

Next, for more flexibly constructing a beampattern toward MN, each BS adopts a two-dimensional array of *M* antennas while each MN has a single antenna for transmission. Given that, the received signal at the MN associated with *i*-th BS would be

(1) $$y_{i}=h_{i,i}f_{i}x_{i}+\sum_{j\neq i}h_{i,j}f_{j}x_{j}+n_{i}$$

In the above, $x_{i},x_{j}\in C$ are the transmitted signals form the *i*-th and *j*-th BSs, complying with the power constraint $E\{|x_{i}\left|\right\}=P_{i}$ and $E\{|x_{j}\left|\right\}=P_{j}$, where $P_{i}$ and $P_{j}$ are the transmit powers of the *i*-th and *j*-th BSs. In addition, $h_{i,i},h_{i,j}\in C^{M\times 1}$ are the channel vectors from the *i*-th and *j*-th BSs to the MN at the *i*-th BS, and $f_{i},f_{j}\in C^{M\times 1}$ denote the downlink beamforming vectors adopted at the *i*-th BS and *j*-th BSs, respectively. As the last term, $n_{i}$ represents the noise at the receiver sampled from a complex normal distribution with zero mean and variance $\sigma_{n}^{2}$.

**Beamforming:** As mentioned previously, for the high propagation loss, analog beamforming vectors are assumed for transmission, and each $f_{i},i=1,2,\cdots ,\left|F\right|$, consists of the beamforming weights for a two-dimensional (2D) planar array steered towards MN. More specifically, let each BS have a 2D array of antennas in the *x*–*y* plane, in which the antenna *m* is located at

(2) $$d_{m}=\left(a_{m}\lambda ,b_{m}\lambda \right)$$

where λ is the wavelength. Given the elevation direction $\psi_{d}$ and the azimuthal direction $\phi_{d}$, the phased weights for the 2D array steered towards the angle $\left(\psi_{d},\phi_{d}\right)$ in the polar coordinates can be given by $e^{-j2\pi \sin\psi_{d}\left(a_{m}\cos\phi_{d}+b_{m}\sin\phi_{d}\right)}$. If the target is located on the *x*–*y* plane, $\sin\psi_{d}$ will be 1 and the weights can be simplified as $e^{-j2\pi (a_{m}\cos\phi_{d}+b_{m}\sin\phi_{d})}$. Given that, we consider every beamforming vector to be selected from a steering-based beamforming codebook F with |F| elements, wherein the *n*-th element or the array steering vector in the direction $\phi_{n}$ is given by

(3) $$\begin{array}{ccc} & & \;f_{n}\overset{▵}{=}a\left(\phi_{n}\right)=\frac{1}{\sqrt{M}}\left[1,e^{-j2\pi (a_{1}\cos\phi_{n}+b_{1}\sin\phi_{n})},\cdots ,e^{-j2\pi (a_{M-1}\cos\phi_{n}+b_{M-1}\sin\phi_{n})}\right] \end{array}$$

### 2.2. Channel Model

With the beamforming vector introduced above, we consider a narrow-band geometric channel model which is widely used for mmWave networks [52,53,54]. Specifically, the channel from BS *i* to the MN in BS *j* is formulated here as

(4) $$h_{i,j}=\frac{\sqrt{M}}{\rho_{i,j}}\sum_{p=1}^{N_{i,j}^{p}}\alpha_{i,j}^{p}a\left(\phi_{i,j}^{p}\right)$$

where $\rho_{i,j}$ represents the path-loss between BS *i* and the MN associated with BS *j*. $\alpha_{i,j}^{p}$ is the complex path gain. $a(\phi_{i,j}^{p})$ denotes the array response vector with respect to $\phi_{i,j}^{p}$, which is the angle of departure (AoD) of the *p*-th path. $N_{i,j}^{p}$ is the number of channel paths, and when compared with those for sub-6G, the number for mmWave is usually a small number [55,56]. Next, let the received power measured by the MN associated with BS *i* over a set of resource blocks (RBs) on the channel from BS *j* to the MN be $P_{j}{\left|h_{i,j}f_{j}\right|}^{2}$. Given that, the received signal to noise and interference ratio (SINR) for the MN associated with BS *i* can be obtained by

(5) $$\frac{P_{i}{\left|h_{i,i}f_{i}\right|}^{2}}{\sum_{j\neq i}P_{j}{\left|h_{j,i}f_{j}\right|}^{2}+\sigma_{n}^{2}}$$

As shown above, each BS *i* uses $P_{i}$ to transmit to its user with beamforming vector $f_{i}$. When incorporating SWIPT into power allocation, the use of beamforming on the mmWave MIMO system provides a new solution to resolve both interference and energy problems [57,58,59]. To this end, each MN in the network is installed with a power splitting unit to split the received signal for information decoding and energy harvesting simultaneously. Given that, the beamforming would provide a dedicated beam for MN through which power control and power splitting for energy harvesting can be realized at the same time. More specifically, in the power splitting architecture for downlink, the received signal at the MN associated with BS *i* which transmits with its beamforming vector $f_{i}$, and transmit power $P_{i}$ is split into two separate signal streams according to the power split ratio $\theta_{i}$, which will be determined in the sequel to maximize the system utility. In addition, when the technology of successive interference cancellation (SIC) is employed to mitigate the interference for data decoding, the stronger signal would be decoded first, and the weaker signals remaining could contribute to the interferences for decoding. With P and F to denote the sets for the transmit power and the power split ratio, respectively, in addition to the above, the SINR at the received MN *i* with SWIPT and SIC could be obtained by

(6) $$\begin{array}{ccc} & & \;\gamma_{i}\left(P,\theta_{i},F\right)=\left(1-\theta_{i}\right)\frac{P_{i}{\left|h_{i,i}f_{i}\right|}^{2}}{\overset{\sum }{{}_{{}_{j\neq i,P_{i}|h_{i,i}f_{i}{|}^{2}>P_{j}{\left|h_{j,i}f_{j}\right|}^{2}}}}P_{j}{\left|h_{j,i}f_{j}\right|}^{2}+\sigma_{n}^{2}} \end{array}$$

As shown above, $1-\theta_{i}$ denotes the fraction of signal for the data transmission of SWIPT. In addition, with SIC [60], when there are multiple signals received by the MN associated with BS *i* concurrently, it will decode the stronger signal, and treat the weaker signals as interference. Here, if there are stronger signals from some BSs, they would be decoded and deleted first. Then, the desired signal will be obtained by treating the weaker signals from the other BSs if they exist, noted here by $j\neq i,P_{i}|h_{i,i}f_{i}{|}^{2}>P_{j}{\left|h_{j,i}f_{j}\right|}^{2}$, as the interference for decoding in addition to the noise $\sigma_{n}^{2}$.

### 2.3. Problem Formulation

Providing these essential models, our aim is to jointly optimize beamforming vectors, transmit powers, and power split ratios at the BSs to make the best trade-off between data rates, harvested energies, and power consumption from all MNs served in the SWIPT-enabled network with SIC, which is formulated as a complex multiple resource allocation (MRA) optimization problem subject to different allocation constraints that resulted from the different types of resources involved, shown as follows:

(7a) $$\begin{array}{cccc} \left(P1\right)\overset{{}_{\max}}{{}_{P_{i},\theta_{i},f_{i},\forall i}} & \; & \sum_{i}U_{i}\left(P,\theta_{i},F\right) & \end{array}$$

(7b) $$\begin{array}{ccc} \mathrm{subject}\mathrm{to} & P_{min}\leq P_{i}\leq P_{max}, & \;\;\;\;\;\forall i \end{array}$$

(7c) $$\begin{array}{cc} 0\leq \theta_{i}\leq 1, & \;\;\;\;\;\forall i \end{array}$$

(7d) $$\begin{array}{cc} f_{i}\in F, & \;\;\;\;\;\forall i \end{array}$$

where $U_{i}\left(P,\theta_{i},F\right)$ in (7a) denotes the utility function for the trade-off to be introduced in (19). (7b) specifies the constraint that the transmit power, $P_{i}$, should be ranged between the minimum transmit power, $P_{min}$, and the maximum transmit power, $P_{max}$. (7c) requires $\theta_{i}$ to be a nonnegative ratio number no larger than 1. Finally, (7d) says that the vector, $f_{i}$, should be selected from its codebook F.

Clearly, if $U_{i}$ in the objective involves $\gamma_{i}$ in (6), (P1) will be a mixed integer nonlinear programming (MINLP) problem. It would be even a non-convex MINLP problem due to the non-convexity of the objective function and the allocation constraints involving discrete values, and its solution is hard to find even using an optimization tool. To resolve this hard problem efficiently, we propose two kinds of innovative approaches based on deep reinforcement learning, game theory, or both, resulting in data-driven, model-driven, or hybrid iterative algorithms which could be operated in a single layer or two different layers, as introduced in the following. In addition, for clarity, we summarize the import symbols for the approaches to be introduced in Table A1 located in Appendix A due to its size.

## 3. Single-Layer Learning-Based Approaches

Determining an exact state transition model for (P1) through a model-based dynamic programming algorithm is challenging because the MRA problem on transmit power, power split ratio, and beamforming vector is location dependent. It is not trivial to list all the state–action pairs to be found in a state transition model predefined. Therefore, we design two single-layer learning-based algorithms derived from Markov decision process (MDP) to resolve this problem.

### 3.1. Q-Learning Approach

The Q-learning algorithm is based on the MDP that can be defined as a 4-tuple <$\tilde{S},\tilde{A},\tilde{R},\tilde{P}$>, where $\tilde{S}=\left\{s_{1},s_{2},\cdots ,s_{m}\right\}$ is the finite set of states, and $\tilde{A}=\left\{a_{1},a_{2},\cdots ,a_{n}\right\}$ is the set of discrete actions. $\tilde{R}\left(s,a,s^{\prime }\right)$ is the function to provide reward r defined at state $s\in \tilde{S}$, action $a\in \tilde{A}$, and next state $s^{\prime }$. ${\tilde{P}}_{ss^{\prime }\left(a\right)}=p\left(s^{\prime }|s,a\right)$ is the transition probability of the agent at state *s* taking action *a* to migrate to state $s^{\prime }$. Given that, reinforcement learning is conducted to find the optimal policy $\pi^{*}\left(s\right)$ that can maximize the total expected discounted reward. Among the different approaches to this end, Q-learning is widely considered, which adopts a value function $V^{\pi }\left(s\right)\to r$ for the expected value to be obtained by policy π from each $s\in \tilde{S}$. Specifically, based on the infinite horizon discounted MDP, the value function in the following is formulated to show the goodness of π as

(8) $$V^{\pi }\left(s\right)=E_{\pi }\left\{\sum_{k=0}^{\infty }\zeta^{k}r_{i}^{k+1}|s^{0}=s\right\}$$

where 0≤ζ≤1 denotes the discount factor, and E is the expectation operation. Here, the optimal policy is defined to map the states to the optimal action in order to maximize the expected cumulative reward. In particular, the optimal action at each sate *s* can be obtained with the Bellman equation [61]:

(9) $$V^{*}\left(s\right)=V^{\pi^{*}}=\max_{a\in \tilde{A}}\left\{E\left(r\left(s,a\right)+\zeta \sum_{s^{\prime }\in \tilde{S}}P_{ss^{\prime }}V^{*}\left(s^{\prime }\right)\right)\right\}$$

Given that, the action–value function is in fact the expected reward of this model starting from state *s* which takes action *a* according to policy π; that is,

(10) $$Q^{\pi }\left(s,a\right)=E\left(r(s,a)\right)+\zeta \sum_{s^{\prime }\in \tilde{S}}P_{ss^{\prime }}V\left(s^{\prime }\right)$$

Let the optimal policy $Q^{*}\left(s,a\right)$ be $Q^{\pi^{*}}$. Then, we can obtain

(11) $$V^{*}\left(s\right)=\max_{a\in \tilde{A}}Q^{*}\left(s,a\right)$$

The strength of Q-learning can now be revealed as it can learn $\pi^{*}$ without knowing the environment dynamics or $P_{ss^{\prime }}\left(a\right)$, and the agent can learn it by adjusting the Q value with the following update rule:

(12) $$Q\left(s_{t},a_{t}\right)=\left(1-\alpha \right)Q\left(s_{t},a_{t}\right)+\alpha \left[r_{t}+\zeta \max_{a^{\prime }\in \tilde{A}}Q\left(s^{\prime },a^{\prime }\right)\right]$$

where α∈[0,1) denotes the learning rate.

Given this strength, the application of Q-learning is, however, limited because the optimal policy can be obtained only when the state-action spaces are discrete and the dimension is relatively small. Fortunately, after considerable investigations on the deep learning techniques, reinforcement learning has made significant progress to replace a Q-table with the neural network, leading to DQN that can approximate $Q(s_{t},a_{t})$. In particular, in DQN, the Q value in time *t* is rewritten as $Q(s_{t},a_{t},\omega )$ wherein ω is the weight of a deep neural network (DNN). Given that, the optimal policy $\pi^{*}\left(s\right)$ in DQN can be represented by $\pi^{*}\left(s\right)=\arg\max_{a^{\prime }}Q^{*}\left(s_{t},a^{\prime },\omega \right)$, where $Q^{*}$ denotes the optimal Q value obtained through DNN. The goal of this approach is then to choose the approximated action $a_{t+1}=\pi^{*}\left(s_{t+1}\right)$, and the approximated Q value is given by

(13) $$\hat{Q}\left(s_{t},a_{t},\omega^{\prime }\right)=r\left(s_{t},a_{t},\omega^{\prime }\right)+\zeta \max_{a^{\prime }\in \tilde{A}}\left[Q(s_{t+1},a^{\prime },\omega )\right]$$

In the above, ω will be updated by minimizing the loss function:

(14) $$\hat{L}=E\left[{\left(\hat{Q}\left(s_{t},a_{t},\omega^{\prime }\right)-Q\left(s_{t+1},a_{t+1},\omega^{\prime }\right)\right)}^{2}\right]$$

**Deep Q learning elements:** Following the Q-learning design approach, we next define state, action, and reward function specific for solving (P1) as follows:

(1) **State:** First, if there are *n* links in the network, the state at time *t* is represented in the sequel by using the capital notations for their components and using the superscript such as “(t)” for the time index as follows:
  (15) $$s^{\left(t\right)}=\left\{L^{\left(t\right)},P^{\left(t\right)},\Theta^{\left(t\right)},F^{\left(t\right)}\right\}$$
  where $L^{\left(t\right)}=\left\{L_{1}^{\left(t\right)},\cdots ,L_{n}^{\left(t\right)}\right\}$, $P^{\left(t\right)}=\left\{P_{1}^{\left(t\right)},\cdots ,P_{n}^{\left(t\right)}\right\}$, $\Theta^{\left(t\right)}=\left\{\theta_{1}^{\left(t\right)},\cdots ,\theta_{n}^{\left(t\right)}\right\}$, and $F^{\left(t\right)}=\left\{f_{1}^{\left(t\right)},\cdots ,f_{n}^{\left(t\right)}\right\}$. In the above, $L_{i}^{\left(t\right)}=\left(X_{i}^{\left(t\right)},Y_{i}^{\left(t\right)}\right)$ denotes the Cartesian coordinates of MN in link *i* at time *t*, while the others, i.e., $P_{i}^{\left(t\right)}$, $\theta_{i}^{\left(t\right)}$, and $f_{i}^{\left(t\right)}$, denote the transmit power, power splitting ratio, and beamforming vector for link *i* at time *t*, respectively.
  Among these variables, the transmit power is usually the only parameter to be considered in many previous works [27,62]. In the complex MRA problem also involving other types of resources, it is still a major factor affecting the system performance based on SINR in (5) that would be significantly impacted by the power, and thus we consider two different state formulations for $P^{\left(t\right)}$ as follows.
  - *Power state formulation 1 (PSF1):* First, to align with the industry standard [33] which chooses integers for power increments, we consider a ±1 dB offset representation similar to that shown in [51], as the the first formulation for the power state. Specifically, given an initial value $P_{i}^{0}$, the transmit power $P_{i},\forall i$ (despite *t*), will be chosen from the set
    (16) $$\begin{array}{ccc} & & \;P_{i}^{1}\overset{▵}{=}\left\{10^{-0.1\cdot K_{min}}P_{i}^{0},\cdots ,10^{-0.1}P_{i}^{0},P_{i}^{0},10^{0.1}P_{i}^{0},\cdots ,10^{0.1\cdot K_{max}}P_{i}^{0}\right\} \end{array}$$
    where $K_{{}_{min}}$=$\lfloor -10\log_{{}_{10}}\left(\frac{P_{{}_{min}}}{P_{i}^{0}}\right)\rfloor$ and $K_{{}_{max}}$=$\left\lfloor 10\log_{{}_{10}}\left(\frac{P_{{}_{max}}}{P_{i}^{0}}\right)\right\rfloor$.
  - *Power state formulation 2 (PSF2):* Next, as shown in [27], the performance of a power-controllable network can be improved by quantizing the transmit power through a logarithmic step size instead of linear step size. Given that, the transmit power $P_{i},\forall i$ could be selected from the set
    (17) $$P^{2}\overset{▵}{=}\left.\left\{P_{min}{\left(\frac{P_{min}}{P_{max}}\right)}^{\frac{j}{|P^{2}|-2}}\right|j=0,\cdots ,\left|P^{2}\right|-2\right\}$$
  Apart from the above, the other parameters, such as $\theta_{i},\forall i$, can be chosen from the splitting ratio set Θ with linear step size, and $f_{i},\forall i$ can be selected from the predefined codebook F with |F| finite vectors or elements.
(2) **Action:** The action of this process at time *t*, $a^{\left(t\right)}$ is selected from a set of binary decisions on the variables
  (18) $$\hat{A}=\left\{{\hat{A}}_{P},{\hat{A}}_{\Theta },{\hat{A}}_{F}\right\}$$
  where ${\hat{A}}_{P}={\hat{A}}_{p_{1}}\times {\hat{A}}_{p_{2}}\cdots \times {\hat{A}}_{p_{n}}\in {\left\{\pm 1\right\}}^{n}$, ${\hat{A}}_{\Theta }={\hat{A}}_{\theta_{1}}\times {\hat{A}}_{\theta_{2}}\cdots \times {\hat{A}}_{\theta_{n}}\in {\left\{\pm 1\right\}}^{n}$, and ${\hat{A}}_{F}={\hat{A}}_{f_{1}}\times {\hat{A}}_{f_{2}}\cdots \times {\hat{A}}_{f_{n}}\in {\left\{\pm 1\right\}}^{n}$ denote all the possible binary decisions on the three types of variables involved, respectively. That is, the agent can decide each link *i* to increase or decrease each of the variables to the next quantized value according to ${\hat{A}}_{p_{i}}^{\left(t\right)}$, ${\hat{A}}_{\theta_{i}}^{\left(t\right)}$ and ${\hat{A}}_{f_{i}}^{\left(t\right)}$ in $a^{\left(t\right)}$, respectively.
  Note that, as the number of values of a variable is limited, when reaching the maximum or minimum value with a binary action chosen from $\hat{A}$, a modulo operation is used to decide the index for the next quantized value in the state space. For example, in PSF2, if $P_{i}^{\left(t\right)}=P_{min}{\left(\frac{P_{min}}{P_{max}}\right)}^{\frac{j}{|P^{2}|-2}}$ with j=0, and $j+{\hat{A}}_{p_{i}}^{\left(t\right)}<0$, then the modulo operation will lead to $P_{i}^{\left(t+1\right)}=P_{min}{\left(\frac{P_{min}}{P_{max}}\right)}^{\frac{j^{\prime }}{|P^{2}|-2}}$ with $j^{\prime }=\left|P^{2}\right|-2$ in $P^{2}$. As another example, with $f_{min}=1$ and $f_{max}=\left|F\right|$ to denote the first and the last vector in the codebook F, respectively, the action of increasing or decreasing $f_{min}\leq f_{i}^{\left(t\right)}\leq f_{max}$ by 1 will choose the previous or the next vector of $f_{i}^{\left(t\right)}$ in F as $f_{i}^{\left(t+1\right)}$, and a similar modulo operation will also be applied to keep $f_{i}^{\left(t+1\right)}$ within $[f_{min}$, $f_{max}]$.
(3) **Reward:** To reduce the power consumption for green communication while maintaining the desired trade-off among the data rate and the energy harvesting, we introduce a reward function that can represent a trade-off among the three metrics properly normalized for link *i* with parameters $\lambda_{i}$, $\mu_{i}$, and $\nu_{i}$, at time *t*, as
  (19) $$\begin{array}{ccc} & & \;U_{i}\left(P^{\left(t\right)},\theta_{i}^{\left(t\right)},F^{\left(t\right)}\right)=\lambda_{i}r_{i}\left(P^{\left(t\right)},\theta_{i}^{\left(t\right)},F^{\left(t\right)}\right)+\mu_{i}E_{i}\left(P^{\left(t\right)},\theta_{i}^{\left(t\right)},F^{\left(t\right)}\right)-\nu_{i}P_{i}^{\left(t\right)} \end{array}$$
  where $r_{i}\left(P^{\left(t\right)},\theta_{i}^{\left(t\right)},F^{\left(t\right)}\right)$ denotes the data rate of link *i* obtained at time *t*, which can be represented by
  (20) $$r_{i}\left(P^{\left(t\right)},\theta_{i}^{\left(t\right)},F^{\left(t\right)}\right)=\log\left(1+\gamma_{i}\left(P^{\left(t\right)},\theta_{i}^{\left(t\right)},F^{\left(t\right)}\right)\right)$$
  In addition, $E_{i}\left(P^{\left(t\right)},\theta_{i}^{\left(t\right)},F_{i}^{\left(t\right)}\right)$ is the energy harvested at MN of link *i* at time *t*, represented in the log scale as
  (21) $$E_{i}\left(P^{\left(t\right)},\theta_{i}^{\left(t\right)},F^{\left(t\right)}\right)=\log\left(e_{i}\left(P^{\left(t\right)},\theta_{i}^{\left(t\right)},F^{\left(t\right)}\right)\right)$$
  wherein the harvested energy in its raw form is given by
  (22) $$\begin{array}{ccc} & & \;e_{i}\left(P^{\left(t\right)},\theta_{i}^{\left(t\right)},F^{\left(t\right)}\right)=\theta_{i}\delta \left(P_{i}^{\left(t\right)}|h_{i,i}^{\left(t\right)}f_{i}^{\left(t\right)}{|}^{2}+\sum_{j\neq i}P_{j}^{\left(t\right)}{\left|h_{j,i}^{\left(t\right)}f_{j}^{\left(t\right)}\right|}^{2}+\sigma_{n}^{2}\right) \end{array}$$
  In the above, δ is the power conversion efficiency, and $\nu_{i}$ is the price or cost for the power consumption $P_{i}^{\left(t\right)}$ to be paid for link *i*’s transmission. Note that the log representation is considered here to accommodate a normalization process in deep learning similar to the batch normalization in [63]. Otherwise, the data rate $r_{i}\left(P^{\left(t\right)},\theta_{i}^{\left(t\right)},F^{\left(t\right)}\right)$ obtained with a log operation and the raw energy harvesting $e_{i}\left(P^{\left(t\right)},\theta_{i}^{\left(t\right)},F^{\left(t\right)}\right)$ without the (log) operation may be directly combined in the utility function. If so, with the metric values lying in very different ranges, such a raw representation could cause problems in the training process. Note also that, although $\lambda_{i}$ and $\mu_{i}$ could be set to compensate the scale differences, a very high energy obtained in certain case can still happen to significantly vary the utility function and impede the learning process. By taking these into account, the system utility at time *t* can be represented by the sum of these link rewards as
  (23) $$U^{\left(t\right)}=U\left(P^{\left(t\right)},\Theta^{\left(t\right)},F^{\left(t\right)}\right)=\sum_{i}U_{i}\left(P^{\left(t\right)},\theta_{i}^{\left(t\right)},F^{\left(t\right)}\right)$$

**Policy selection:** In general, Q-learning is an off-policy algorithm that can find a suboptimal policy even when its actions are obtained from an arbitrary exploratory selection policy [64]. Following that, we conduct the DQN-based MRA algorithm to have a near-greedy action selection policy, which consists of (1) exploration mode and (2) exploitation mode. On the one hand, in exploration mode, the DQN agent would randomly try different actions at every time *t* for getting a better state-action or Q value. On the other hand, in exploitation mode, the agent will choose at each time *t* an action $a^{\left(t\right)}$ that can maximize the Q value via DNN with weight ω; that is, $a^{\left(t\right)}=\arg\max_{a^{\prime }\in A}Q^{*}\left(s_{t},a^{\prime },\omega \right)$. More specifically, we conduct the agent to explore with a probability ϵ and to exploit with a probability 1−ϵ, where ϵ∈(0,1) denotes a hyperparameter to adjust the trade-off between exploration and exploitation, resulting in a ϵ-greedy selection policy.

**Experience replay:** This algorithm also includes a buffer memory *D* as a replay memory to store transactions $\left(s^{\left(t\right)},a^{\left(t\right)},r^{\left(t\right)},s^{\prime }\right)$, where reward $r^{\left(t\right)}=U^{\left(t\right)}$ is obtained by (23) at time *t*. Given that, at each learning step, a mini-batch is constructed by randomly sampling the memory pool and then a stochastic gradient descent (SGD) is used to update ω. By reusing the previous experiences, the experience replay makes the stored samples to be exploited more efficiently. Furthermore, by randomly sampling the experience buffer, a more independent and identically distributed data set could be obtained for training.

As a summary of these key points introduced above, we formulate the single-layer DQN-based MRA training algorithm with a pseudo code representation shown in Algorithm 1 for easy reference.
**Algorithm 1** The single-layer DQN-based MRA training algorithm.1:(Input) $\lambda_{i},\mu_{i},\nu_{i},\forall i$, batch size η, learning rate α, minimum exploration rate $\epsilon_{min}$, discount factor ζ, and exploration decay rate *d*;2:(Output) Learned DQN to decide $P_{i},\theta_{i},f_{i},\forall i$, for (7);3:Initialize action $a^{\left(0\right)}$ and replay buffer D=∅;4:**for** episode = 1 to M **do**5:    Initialize state $s^{\left(0\right)}$;6:    **for** time t=1 to N **do**7:        Observe current state $s^{\left(t\right)}$;8:        $\epsilon =\max(\epsilon \cdot d,\epsilon_{min})$;9:        **if** random number r<ϵ **then**10:           Select $a^{\left(t\right)}\in \hat{A}$ at random;11:        **else**12:           Select $a^{\left(t\right)}=\arg\max_{a^{\prime }}Q^{*}\left(s^{\left(t\right)},a^{\prime },\omega \right)$;13:        **end if**14:        Observe next state $s^{\prime }$;15:        Store transition $\left(s^{\left(t\right)},a^{\left(t\right)},r^{\left(t\right)},s^{\prime }\right)$ in *D*, where $r^{\left(t\right)}$ is $U^{\left(t\right)}$ obtained with (23);16:        Select randomly η stored samples $\left(s^{\left(j\right)},a^{\left(j\right)},r^{\left(j\right)},s^{\left(j+1\right)}\right)$ from *D* for experience;17:        Obtain $\hat{Q}\left(s^{\left(j\right)},a^{\left(j\right)},\omega^{\prime }\right)$ for all *j* samples with (13);18:        Perform SGD to minimize the loss in (14) for finding the optimal weight of DNN, $\omega^{*}$;19:        Update $\omega =\omega^{*}$ in the DQN;20:        $s^{\left(t\right)}=s^{\prime }$;21:    **end for**22:**end for**

### 3.2. DDPG-Based Approach

Similar to that found in the literature [28,29], as a deep reinforcement learning algorithm, DQN would be superior to the classical Q-learning algorithm because it can handle the problems with high-dimensional state spaces that can hardly be done with the former. However, DQN still works on a discrete action space, and suffers the curse of dimensionality when the action space becomes large. For this, we next develop a deep deterministic policy gradient (DDPG)-based algorithm that can find optimal actions in a continuous space to solve this MRA optimization problem without quantizing the actions that should be done for the DQN-based algorithm.

Specifically, with DDPG, we aim to determine an action *a* to maximize the action–value function Q(s,a) for a given state *s*. That is, our goal is to find

(24) $$a^{*}\left(s\right)=\arg\max_{a}Q\left(s,a\right)$$

as that done with DQN introduced previously. However, unlike DQN, there are two neural networks for DDPG, namely actor network and critic network, and each contains two subnets, namely online net and target net, with the same architecture. First, the actor network with the weight of DNN, $\omega_{a}$, which is called “actor parameter”, will take state *s* to output a deterministic action *a*, denoted by $Q_{a}\left(s;\omega_{a}\right)$. Second, the critic network with the weight of DNN, $\omega_{c}$, which is called “critic parameter” will take state *s* and *a* as its inputs to produce the state–value function, denoted by $Q(s,a;\omega_{c})$, to simulate a table for Q-learning or Q-table that would get rid of the curse of dimensionality. Given that, two key features of DDPG can be summarized as follows:

(1) *Exploration*: As defined, the actor network is conducted to provide solutions to the problem, playing a crucial role in DDPG. However, as it is designed to produce only deterministic actions, additional noise, *n*, is added to the output so that the actor network can explore the solution space. That is,
  (25) $$a\left(s\right)=Q_{a}\left(s;\omega_{a}\right)+n$$
(2) *Updating the networks*: Next, with the notation $\left(s,a,r,s^{\prime }\right)$ to denote the transaction wherein reward r is obtained by taking action *a* at state *s* to migrate to $s^{\prime }$ as that in DQN, the update procedures for the critic and actor networks can be further summarized in the following.
  - As shown in (24), the actor network is updated by maximizing the state–value function. In terms of the parameters $\omega_{a}$ and $\omega_{c}$, this maximization problem can be rewritten to find $J\left(\omega_{a}\right)=Q\left(s,a;\omega_{c}\right){|}_{a=Q_{a}\left(s;\omega_{a}\right)}$. Here, as the action space is continuous and the state–value function is assumed to be differentiable, the actor parameter, $\omega_{a}$, would be updated by using the gradient ascent method. Furthermore, as the gradient depends on the derivative of the objective function with respect to $\omega_{a}$, the chain rule can be applied as
    (26) $$\nabla \omega_{a}J\left(\omega_{a}\right)=\nabla_{a}Q\left(s,a;\omega_{c}\right){|}_{a=Q_{a}\left(s;\omega_{a}\right)}\nabla \omega_{a}Q_{a}\left(s;\omega_{a}\right)$$
    Then, as the actor network would output $Q_{a}\left(s;\omega_{a}\right)$ to be the action adopted by the critic network, the actor parameter $\omega_{a}$ can be updated by maximizing the critic network’s output with the action obtained from the actor network, while fixing the critic parameter $\omega_{c}$.
  - Apart from the actor network to generate the needed actions, the critic network is also crucial to ensure that the actor network is well trained. To update the critic network, there are two aspects to be considered. First, with $Q_{a^{\prime }}\left(s;\omega_{a^{\prime }}\right)$ from the target actor network to be an input of the target critic network, the state–value function would produce
    (27) $$y=r+\zeta \bar{Q}\left(s^{\prime },a;\omega_{c}\right){|}_{a=Q_{a^{\prime }}\left(s;\omega_{a^{\prime }}\right)}$$
    Second, the output of the critic network, $Q(s,a;\omega_{c})$, can be regarded as another source to estimate the state–value function. Based on these aspects, the critic network can be updated by minimizing the following loss function:
    (28) $$\hat{L}={\left(y-Q\left(s,a;\omega_{c}\right)\right)}^{2}$$
    Given that, the critic parameter, $\omega_{c}$, can be obtained by finding the parameter to minimize this loss function.
  - Finally, the target nets in both critic and actor networks can be updated with the soft update parameter, τ, on their parameters $\omega_{c}^{\prime }$ and $\omega_{a}^{\prime }$, as follows:
    (29) $$\omega_{c}^{\prime }=\tau \omega_{c}+\left(1-\tau \right)\omega_{c}^{\prime },\;\omega_{a}^{\prime }=\tau \omega_{a}+\left(1-\tau \right)\omega_{a}^{\prime }$$

**Action representation for the MRA problem:** As defined, the actor network outputs the deterministic action $a^{*}=Q_{a}\left(s;\omega_{a}\right)$. Due to the deterministic, a dynamic ϵ-greedy policy is used to determine the action by adding a noise term $n^{\left(t\right)}$ to explore the action space. Here, as the state of this work involves different types of variables, the action resulting at time *t* in fact consists of three parts as $a^{{\left(t\right)}^{*}}=\left\{A_{P}^{{\left(t\right)}^{*}},A_{\Theta }^{{\left(t\right)}^{*}},A_{F}^{{\left(t\right)}^{*}}\right\}$. When added with the corresponding noises, the exploration action $a^{\left(t\right)}$ would be specified as

(30) $$\begin{array}{ccc} & & \;a^{\left(t\right)}=\left[{\left[A_{P}^{{\left(t\right)}^{*}}+n_{P}^{\left(t\right)}\right]}_{P_{low}}^{P_{up}},{\left[A_{\Theta }^{{\left(t\right)}^{*}}+n_{\Theta }^{\left(t\right)}\right]}_{\Theta_{low}}^{\Theta_{up}},{\left[A_{F}^{{\left(t\right)}^{*}}+n_{F}^{\left(t\right)}\right]}_{F_{low}}^{F_{up}}\right] \end{array}$$

where the different parts of $a^{\left(t\right)}$ are clipped to the intervals $\left[x_{up},x_{low}\right],x\in \left\{P,\Theta ,F\right\}$, according to the different types of variables, and the added noises are obtained with a normal distribution also based on the different types as

(31) $$n_{x}^{\left(t\right)}∼N\left(0,d^{\left(t\right)}\left(x_{up}-x_{low}\right)\right)$$

where $d^{\left(t\right)}$ denotes the exploration decay rate at time *t*.

**State normalization and quantization:** As shown in the previous works [32,63,65], a state normalization to preprocess the training sample sets would lead to a much easier and faster training process. In our work, the three types of variables, $P^{\left(t\right)},\Theta^{\left(t\right)}$, and $F^{\left(t\right)}$ (shown in vector forms) in $s^{\left(t\right)}$ may have their values lying in very different ranges, which could cause problems in a training process. To prevent them, we normalize the coordinates with the cell radius, and these variables with the scale factors $\varsigma_{1}$, $\varsigma_{2}$, and $\varsigma_{3}$, as

(32) $$\;P_{i}^{\left(t\right)}=\varsigma_{1}\frac{{\tilde{P}}_{i}^{\left(t\right)}}{P_{max}},\theta_{i}^{\left(t\right)}=\varsigma_{2}\frac{{\tilde{\theta }}_{i}^{\left(t\right)}}{\theta_{max}},f_{i}^{\left(t\right)}=\varsigma_{3}\frac{{\tilde{f}}_{i}^{\left(t\right)}}{f_{max}},\forall i$$

In the above, $\tilde{f_{i}}$ is an integer variable rounded from its real counterpart to denote which element in the codebook F to be used because the output of DDGP is a continuous action. Specifically, given $a^{\left(t\right)}$=$\left\{a_{P}^{\left(t\right)},a_{\Theta }^{\left(t\right)},a_{F}^{\left(t\right)}\right\}$ where $a_{f_{i}}^{\left(t\right)}$$\in a_{F}^{\left(t\right)}$ = ${\left[A_{F}^{{\left(t\right)}^{*}}\;+\;n_{F}^{\left(t\right)}\right]}_{F_{low}}^{F_{up}}\;$ is obtained by (30), its value at time *t* will be

(33) $${\tilde{f}}_{i}^{\left(t\right)}={\left[\lfloor f_{i}^{\left(t\right)}f_{max}/\varsigma_{3}+a_{f_{i}}^{\left(t\right)}\rfloor \right]}_{f_{min}}^{f_{max}}$$

Note that, after the rounding operation (represented here by the floor function), the value may still be out of its feasible range, and thus a modulo operation similar to that for DQN is also applied here to keep it in $\left[f_{min},f_{max}\right]$. For the other types of variables, the corresponding modulo operations are required to keep them in their feasible ranges as well. Still, due to their continuous nature, a rounding operation is avoided. Specifically, with $a_{P_{i}}^{\left(t\right)}\in a_{P}^{\left(t\right)}$ and $a_{\theta_{i}}^{\left(t\right)}\in a_{\Theta }^{\left(t\right)}$, each ${\tilde{P}}_{i}^{\left(t\right)}$ and ${\tilde{\theta }}_{i}^{\left(t\right)}$ at time *t* would be updated by

(34) $$\begin{array}{ccc} & & \;{\tilde{P}}_{i}^{\left(t\right)}={\left[P_{i}^{\left(t\right)}P_{max}/\varsigma_{1}+a_{P_{i}}^{\left(t\right)}\right]}_{P_{min}}^{P_{max}} \end{array}$$

(35) $$\begin{array}{ccc} & & \;{\tilde{\theta }}_{i}^{\left(t\right)}={\left[\theta_{i}^{\left(t\right)}\theta_{max}/\varsigma_{2}+a_{\theta_{i}}^{\left(t\right)}\right]}_{\theta_{min}}^{\theta_{max}}\; \end{array}$$

Apart from the above, the critic network $Q(s_{c},a;\omega_{c})$ is conducted to transfer gradient in learning, which is not involved in action generation. In particular, the critic network evaluates the current control action based on the performance index (23) while the parameters $P^{\left(t\right)},\Theta^{\left(t\right)}$, and $F^{\left(t\right)}$ of *U* in (23) are obtained by the actor network. Apart from these networks, the DDPG-based algorithm also includes an experience replay mechanism as the DQN counterpart. That is, when the experience buffer is full, the current transition $\left(s^{\left(t\right)},a^{\left(t\right)},r^{\left(t\right)},s^{\prime }\right)$ will replace the oldest one in the buffer *D* where reward $r^{\left(t\right)}=U^{\left(t\right)}$, and then the algorithm would randomly choose η stored transitions to form a mini-batch for updating the networks. Given these sampled transitions, the critic network can update its online net by minimizing the loss function represented by

(36) $${\hat{L}}_{\eta }=\frac{1}{\eta }\sum_{i}{\left(y_{i}-Q\left(s_{i},a;\omega_{c}\right)\right)}^{2}$$

where $y_{i}=r_{i}+\zeta \bar{Q}\left(s_{i}^{\prime },a;\omega_{c}\right){|}_{a=Q_{a^{\prime }}\left(s_{i};\omega_{a^{\prime }}\right)}$. Similarly, the actor network can update its online net with

(37) $$\begin{array}{ccc} & & \;\nabla \omega_{a}J_{\eta }\left(\omega_{a}\right)\;=\;\frac{1}{\eta }\sum_{i}\nabla_{a}Q\left(s_{i},a;\omega_{c}\right){|}_{a=Q_{a}\left(s_{i};\omega_{a}\right)}\nabla \omega_{a}Q_{a}\left(s_{i};\omega_{a}\right) \end{array}$$

Finally, we summarize the single-layer DDPG-based MRA training algorithm in Algorithm 2 to be referred to easily.
**Algorithm 2** The single-layer DDPG-based MRA training algorithm.1:(Input) $\lambda_{i},\mu_{i},\nu_{i},\forall i$, batch size η, actor learning rate $\alpha_{a}$, critic learning rate $\alpha_{c}$, decay rate *d*, discount factor ζ, and soft update parameter τ;2:(Output) Learned actor/critic to decide $P_{i},\theta_{i},f_{i},\forall i$, for (7);3:Initialize actor $Q_{a}\left(s;\omega_{a}\right)$, critic $Q(s,a;\omega_{c})$, action $a^{\left(0\right)}$, replay buffer *D*, and set initial decay rate $d^{\left(0\right)}=1$;4:**for** episode = 1 to M **do**5:     Initialize state $s^{\left(0\right)}$ and $\rho^{\left(0\right)}$;6:     **for** time t=1 to N **do**7:          Normalize state $s^{\left(t\right)}$ with (32);8:          Execute action $a^{\left(t\right)}$ in (30), obtain reward $r^{\left(t\right)}=U^{\left(t\right)}$ with (23), and observe new state $s^{\prime }$;9:          **if** replay buffer *D* is not full **then**10:               Store transition $\left(s^{\left(t\right)},a^{\left(t\right)},r^{\left(t\right)},s^{\prime }\right)$ in *D*;11:          **else**12:               Replace the oldest one in buffer *D* with $\left(s^{\left(t\right)},a^{\left(t\right)},r^{\left(t\right)},s^{\prime }\right)$;13:               Set $d^{\left(t\right)}=d^{\left(t-1\right)}\cdot d$;14:               Randomly choose η stored transitions from *D*;15:               Update the critic online network by minimizing the loss function in (36);16:               Update the actor online network with the gradient obtained by (37);17:               Soft update the target networks with their parameters updated by (29);18:               $s^{\left(t\right)}=s^{\prime }$;19:          **end if**20:     **end for**21:   **end for**

## 4. Two-Layer Hybrid Approach Based on Game Theory and Deep Reinforcement Learning

As exhibited above, DDPG can be used for continuous action spaces as well as high-dimensional state spaces, which would overcome the difficulty of DQN which can apply only to discrete action spaces. However, the MRA problem includes both discrete and continuous variables, which requires DDPG to quantize the continuous variables involved to be their discrete counterparts as shown in (33). In addition, as a data driven approach, deep reinforcement learning does not explicitly benefit from an analytic model specific to the problem. To take the advantages from both data-driven and model-driven approaches, we propose in the following a novel approach that consists of two layers, where the lower layer is responsible for the continuous power allocation (PA) and energy harvest splitting (EHS) by using a game-theory-based iterative method, and the upper layer resolves the discrete beam selection problem (BSP) by using a DQN algorithm. That is, if $f_{i},\forall i$, can be given, PA and EHS on $P_{i}$ and $\theta_{i}$ for each link *i* could be decomposed from the objective. Then, we could simplify the MRA problem by reducing (P1) to a BSP sub-problem and a PA/EHS sub-problem. Specifically, the latter (PA/EHS) is given by

(38a) $$\begin{array}{ccc} \left(P2\right)\overset{{}_{\max}}{{}_{P_{i},\theta_{i}}} & \; & U_{i}\left(P,\theta_{i},F\right) \end{array}$$

(38b) $$\begin{array}{ccc} \mathrm{subject}\mathrm{to} & \; & P_{min}\leq P_{i}\leq P_{max} \end{array}$$

(38c) $$\begin{array}{ccc} \; & \; & 0\leq \theta_{i}\leq 1 \end{array}$$

Clearly, if the BSP sub-problem can be solved, the major challenge of this approach would be the PA/EHS sub-problem shown in (P2). Here, even represented by a simpler form, (P2) is still a non-convex problem whose solution for link *i* will depend on the other links j≠i. That is, despite EHS, the PA problem still remains in (P2) that a larger $P_{i}$ would increase SINR of link *i* while reducing those of the other links j≠i in (6), increase energy harvesting in (22), or both, at the cost $\nu_{i}$ for $P_{i}$ in the objective function.

### 4.1. Game Model

To overcome this difficulty, we convert (P2) into a non-cooperative game among the multiple links which could be regarded as self-interesting players and finding its Nash equilibrium (NE) is the fundamental issue to be considered in this game model. On the one hand, a link *i* can be seen as a non-cooperative game player who can choose its own $P_{i}$ and $\theta_{i}$ to make a trade-off so that a larger $P_{i}$ will lead to a higher SINR value in (6) for data rate, a higher value in (22) for energy harvesting, or both on the cost of a higher power consumption, and vice versa. On the other hand, the utility given in (19) can be considered to reduce the power consumption for green communication while maintaining a desired trade-off among the data rate and the energy harvesting. The game-based pricing strategy is thus designed through which BS can require its link to pay a certain price for the power consumption on its transmission. For this, $\lambda_{i}$ can be interpreted as the willingness of player *i* to pay for the data rate, and $\mu_{i}$ as that to pay for the energy harvesting. Given that, each link or player *i* can determine its $P_{i}$ and $\theta_{i}$ based on price $\nu_{i}$ to maximize its own utility, and in this maximization, $\lambda_{i}$, $\mu_{i}$, and $\nu_{i}$ are predetermined values for player *i* and unknown for the others j≠i, as a basis for the non-cooperative game.

### 4.2. Existence of Nash Equilibrium

To ensure the outcome of the non-cooperative game to be effective, we next show this game to have at least one Nash equilibrium. As noted in [66], a Nash equilibrium point represents a situation wherein every player is unilaterally optimal and no player can increase its utility alone by changing its own strategy. Furthermore, according to the game theory fundamental [66], the non-cooperative game admits at least one Nash equilibrium point if (1) the strategy space is a nonempty, compact and convex set, and (2) the utility function is continuous quasiconcave with respect to the action space. In (P2), the utility function $U_{i}$ can be verified to satisfy the above conditions. Specifically, for the first condition, we can note that the transmit power is bounded by $P_{min}$ and $P_{max}$, i.e., $P_{i}\in \left[P_{min},P_{max}\right]$, and the power splitting ratio, $\theta_{i}$, is a real number bounded by 0 and 1. Let $S_{i}$ be the set of all strategies as its strategy space. Then, the strategy space for each link *i* in the proposed game model can be represented by $S_{i}$=$\left\{\left(P_{i},\theta_{i}\right)\in R^{2}|P_{min}\leq P_{i}\leq P_{max},0\leq \theta_{i}\leq 1\right\}$, which is a compact (closed and bounded) convex set as required.

For the second condition, we can derive the partial differential of the utility function with respect to power $P_{i}$ as

(39) $$\frac{\partial U_{i}}{\partial P_{i}}=\frac{\lambda_{i}\left(1-\theta_{i}\right){\left|h_{i,i}f_{i}\right|}^{2}}{R_{i}-\theta_{i}P_{i}{\left|h_{i,i}f_{i}\right|}^{2}}+\frac{\mu_{i}\theta_{i}\delta {\left|h_{i,i}f_{i}\right|}^{2}}{\theta_{i}\delta \left(P_{i}|h_{i,i}f_{i}{|}^{2}+\sum_{j\neq i}P_{j}{\left|h_{j,i}f_{j}\right|}^{2}+\sigma_{n}^{2}\right)}-\nu_{i}$$

where $R_{i}$ is the total received power at link *i*, which accommodates the effect of SIC involved, as shown as follows:

(40) $$R_{i}=\left\{\begin{array}{c} \sigma_{n}^{2}+\sum_{j\neq i}P_{j}|h_{j,i}f_{j}{|}^{2}+P_{i}|h_{i,i}f_{i}{|}^{2},\mathrm{if}P_{i}|h_{i,i}f_{i}{|}^{2}>\sum_{j\neq i}P_{j}{\left|h_{j,i}f_{j}\right|}^{2}\\ \sigma_{n}^{2}+P_{i}{\left|h_{i,i}f_{i}\right|}^{2}\;,\mathrm{otherwise} \end{array}\right.$$

Similarly, we can obtain the partial differential of the utility with respect to $\theta_{i}$ by

(41) $$\begin{array}{ccc} \;\frac{\partial U_{i}}{\partial \theta_{i}} & = & \frac{\lambda_{i}}{1+\gamma_{i}}\frac{\partial \gamma_{i}}{\partial \theta_{i}}+\frac{\mu_{i}\delta \left(P_{i}|h_{i,i}f_{i}{|}^{2}+\sum_{j\neq i}P_{j}{\left|h_{j,i}f_{j}\right|}^{2}+\sigma_{n}^{2}\right)}{\theta_{i}\delta \left(P_{i}|h_{i,i}f_{i}{|}^{2}+\sum_{j\neq i}P_{j}{\left|h_{j,i}f_{j}\right|}^{2}+\sigma_{n}^{2}\right)}\\ & = & \frac{-\lambda_{i}P_{i}{\left|h_{i,i}f_{i}\right|}^{2}}{R_{i}-\theta_{i}P_{i}{\left|h_{i,i}f_{i}\right|}^{2}}+\frac{\mu_{i}}{\theta_{i}} \end{array}$$

Furthermore, from (39) and (41), the second derivative of the utility function with respect to $P_{i}$ and $\theta_{i}$, respectively, can be obtained by    

(42) $$\begin{array}{ccc} \frac{\partial U_{i}^{2}}{\partial P_{i}^{2}} & = & -\lambda_{i}{\left(\frac{\left(1-\theta_{i}\right){\left|h_{i,i}f_{i}\right|}^{2}}{R_{i}-\theta_{i}P_{i}{\left|h_{i,i}f_{i}\right|}^{2}}\right)}^{2}-\mu_{i}{\left(\frac{|h_{i,i}f_{i}{|}^{2}}{P_{i}|h_{i,i}f_{i}{|}^{2}+\sum_{j\neq i}P_{j}{\left|h_{j,i}f_{j}\right|}^{2}+\sigma_{n}^{2}}\right)}^{2}\\ \frac{\partial U_{i}^{2}}{\partial \theta_{i}^{2}} & = & -\lambda_{i}{\left(\frac{P_{i}{\left|h_{i,i}f_{i}\right|}^{2}}{R_{i}-\theta_{i}P_{i}{\left|h_{i,i}f_{i}\right|}^{2}}\right)}^{2}-\mu_{i}{\left(\frac{1}{\theta_{i}}\right)}^{2} \end{array}$$

It is easy to see that both $\frac{\partial U_{i}^{2}}{\partial P_{i}^{2}}$ and $\frac{\partial U_{i}^{2}}{\partial \theta_{i}^{2}}$ are less than or equal to 0, implying that the utility function is convex. In addition, $U_{i}$ is continuous in $P_{i}$. Consequently, the utility functions, $U_{i},\forall i$, all satisfy the required conditions for the existence of at least one Nash equilibrium.

### 4.3. Power Allocation and Energy Harvest Splitting in the Lower Layer

Based on the non-cooperative game model introduced, the associated BS is responsible for deciding the transmit power $P_{i}$ and the power splitting ratio $\theta_{i}$ for link *i*, with the channel state information $h_{i,j}$ and the weights $\lambda_{i}$ and $\mu_{i}$, which can be done by finding its Nash equilibrium. To see this, we note that, as the utility functions $U_{i},\forall i$, are concave down with respect to $\left(P_{i},\theta_{i}\right)$, this decision can be made by using the solution to the system of equations:

(43) $$\frac{\partial U_{1}}{\partial P_{1}}=0,\cdots ,\frac{\partial U_{n}}{\partial P_{n}}=0,\frac{\partial U_{1}}{\partial \theta_{1}}=0,\cdots ,\frac{\partial U_{n}}{\partial \theta_{n}}=0$$

where *n* denotes the number of links in the network.

To solve the system of equations, we propose an iterative algorithm based on the game model, and through the fixed point iteration process, the system of Equation (43) can be solved numerically. Here, by taking the derivative with respect to $P_{i}$ (resp. $\theta_{i}$) and setting the result equal to 0, we can transform the system into a fixed point form for each link *i* that can facilitate its convergence, as follows:

(44) $$\begin{array}{ccc} P_{i} & = & \frac{\lambda_{i}}{\nu_{i}-\frac{\mu_{i}{\left|h_{i,i}f_{i}\right|}^{2}}{{\hat{R}}_{i}+P_{i}{\left|h_{i,i}f_{i}\right|}^{2}}}-\frac{{\hat{R}}_{i}}{\left(1-\theta_{i}\right){\left|h_{i,i}f_{i}\right|}^{2}} \end{array}$$

(45) $$\begin{array}{ccc} \theta_{i} & = & \frac{\mu_{i}R_{i}}{\left(1+\lambda_{i}\right)P_{i}{\left|h_{i,i}f_{i}\right|}^{2}} \end{array}$$

where ${\hat{R}}_{i}$ is an auxiliary variable denoted by

(46) $${\hat{R}}_{i}=\sigma_{n}^{2}+\sum_{j\neq i}P_{j}{\left|h_{j,i}f_{j}\right|}^{2}$$

To show the iterative process more clearly, we denote the transmit power, the total received power, the auxiliary variable, and the power splitting ratio, for link *i* at the *k*-th iteration, by $P_{i}\left[k\right]$, $R_{i}\left[k\right]$, ${\hat{R}}_{i}\left[k\right]$, and $\theta_{i}\left[k\right]$, respectively. Given that, the iterations on $P_{i}$ and $\theta_{i}$ can be shown by the relationships between iterations *k* and k−1 with their results to be bounded by the corresponding maximum and minimum values as follows:

(47) $$P_{i}\left[k\right]={\left[\frac{\lambda_{i}}{\nu_{i}-\frac{\mu_{i}{\left|h_{i,i}f_{i}\right|}^{2}}{{\hat{R}}_{i}\left[k-1\right]+P_{i}\left[k-1\right]{\left|h_{i,i}f_{i}\right|}^{2}}}-\frac{{\hat{R}}_{i}\left[k-1\right]}{\left(1-\theta_{i}\left[k-1\right]\right){\left|h_{i,i}f_{i}\right|}^{2}}\right]}_{P_{min}}^{P_{max}}$$

(48) $$\theta_{i}\left[k\right]={\left[\frac{\mu_{i}R_{i}\left[k-1\right]}{\left(1+\lambda_{i}\right)P_{i}\left[k-1\right]{\left|h_{i,i}f_{i}\right|}^{2}}\right]}_{\theta_{min}}^{\theta_{max}}$$

### 4.4. Beam Selection in the Upper Layer and the Overall Algorithm

With the transmit powers and energy splitting ratios from the lower layer with a low cost, the two-layer hybrid approach is designed to resolve the remaining beam selection problem with a DQN-based algorithm in the upper layer, which would reduce the computational overhead when compared with the DQN approach in Section 3.1 and the DDPG-based approach in Section 3.2. In addition, unlike the previous approaches considering either discrete action space or continuous action space solely, the two-layer approach obtains the variables in their own domains without either approximating the hybrid space by concretization or relaxing it into a continuous set. As a result, the two-layer approach would achieve higher utilities than the others, as exemplified in the experiments.

**Figure 2:**
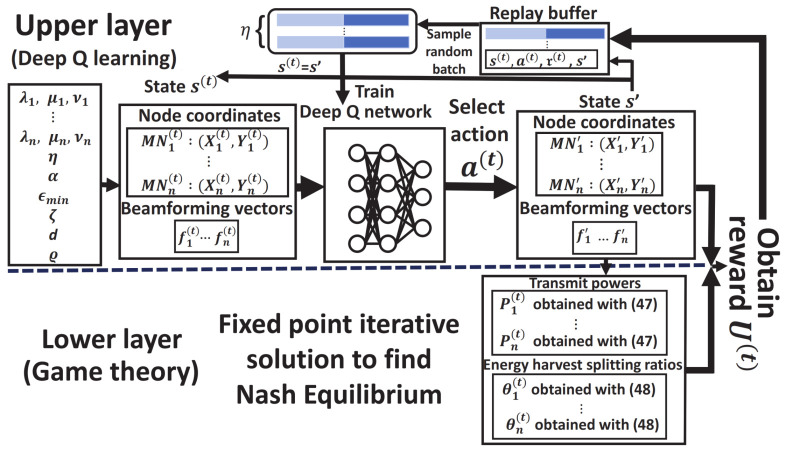
Flowchart of the two-layer hybrid MRA training algorithm. In the upper half, the input and the state (corresponding to line 1 and line 3 in Algorithm 3) are shown by the first box and the second box, respectively (from left to right). After selection (lines 10–14), the new state is shown by the fourth box. In the bottom half, the lower-layer iterations (lines 17–27) are exhibited with a box showing the equations involved. The two halves then cooperatively produce the reward (line 28) shown in the rightmost side toward the remaining boxes at the top denoting the following steps in Algorithm 3.

Specifically, we propose to use a DQN-based algorithm in the upper layer to resolve the beam selection problem in its own discrete action space. When compared with that given in Section 3.1, this algorithm considers locations L and beamforming vectors F only, leading to a reduced DQN model whose state at time *t* is represented by $s^{\left(t\right)}=\left\{L^{\left(t\right)},F^{\left(t\right)}\right\}$, and the action $a^{\left(t\right)}$ is selected from $\hat{A}$ (here including only ${\hat{A}}_{F}$) modified to take into account also the case of no changes. That is, each ${\hat{A}}_{f_{i}}^{\left(t\right)}\in a^{\left(t\right)}$ selected from ${\hat{A}}_{F}$ can now be anyone in $\left\{-1,0,+1\right\}$ instead of ±1, in which 0 implies no changes on the previous beam selection. When the modification integrates with the lower layer, the two-layer hybrid MRA training algorithm has results as shown in Algorithm 3 along with its flowchart shown in Figure 2. Similar to Algorithms 1 and 2, the training algorithm would take the parameters for the utility, the hyperparameters for the learning algorithm, and the parameters for the game-based method, as the input, while producing a learned DQN model as the output that can online decide $P_{i}$, $\theta_{i}$, and $f_{i},\forall i$, for the optimization problem in (7) afterwards. Apart from the input and output, its main steps are summarized as follows:
**Algorithm 3** The two-layer hybrid MRA training algorithm.1:(Input) $\lambda_{i},\mu_{i},\nu_{i},\forall i$, batch size η, learning rate α, minimum exploration rate $\epsilon_{min}$, discount factor ζ, exploration decay rate *d*, and converge threshold ϱ;2:(Output) Learned DQN to decide $P_{i},\theta_{i},f_{i},\forall i$, for (7);3:(Upper-layer DQN-based learning:)4:Initialize action $a^{\left(0\right)}$ and replay buffer D=∅;5:**for** episode = 1 to M **do**6:     Initialize state $s^{\left(0\right)}$;7:     **for** time t=1 to N **do**8:          Observe current state $s^{\left(t\right)}=\left\{L^{\left(t\right)},F^{\left(t\right)}\right\}$;9:          $\epsilon =\max(\epsilon \cdot d,\epsilon_{min})$;10:          **if** random number r<ϵ **then**11:               Select $a^{\left(t\right)}$ from ${\hat{A}}_{F}$ at random;12:          **else**13:               Select $a^{\left(t\right)}=\arg\max_{a^{\prime }}Q^{*}\left(s^{\left(t\right)},a^{\prime },\omega \right)$;14:          **end if**15:          Observe next state $s^{\prime }$;16:          (Lower-layer game-theory-based iteration:)17:          **for** each link *i* **do**18:               **for** iteration k=1 to K **do**19:                    Update $P_{i}\left[k\right]$ with (47);20:                    Update $\theta_{i}\left[k\right]$ with (48);21:                    **if** |$U_{i}\left[k\right]-U_{i}\left[k-1\right]|\leq \varrho$ **then**22:                         $k^{\prime }=k$; break;23:                    **end if**24:               **end for**25:               $k^{*}=\min\left\{k^{\prime },K\right\}$;26:               $P_{i}^{\left(t\right)}=P_{i}\left[k^{*}\right]$; $\theta_{i}^{\left(t\right)}=\theta_{i}\left[k^{*}\right]$;27:          **end for**28:          Determine $U_{i}^{\left(t\right)}$ based on $P_{i}^{\left(t\right)}$ and $\theta_{i}^{\left(t\right)}$ in the lower layer, and $f_{i}^{\left(t\right)}$ in the upper layer, ∀i;29:          Store transition $\left(s^{\left(t\right)},a^{\left(t\right)},r^{\left(t\right)},s^{\prime }\right)$ in *D*;30:          Select η random samples $\left(s^{\left(j\right)},a^{\left(j\right)},r^{\left(j\right)},s^{\left(j+1\right)}\right)$ from *D*;31:          Calculate $\hat{Q}\left(s^{\left(j\right)},a^{\left(j\right)},\omega^{\prime }\right)$ and perform SGD to find the optimal weight of DNN, $\omega^{*}$;32:          Update $\omega =\omega^{*}$ for DQN in the upper layer;33:          $s^{\left(t\right)}=s^{\prime }$;34:   **end for**35:**end for**

- Observe state $s^{\left(t\right)}$ at time *t* for beam section.
- Select an optimal action from $a^{\left(t\right)}$ at time step *t*.
- Given selected beamforming vectors $F^{\left(t\right)}$, obtain transmit powers $P^{\left(t\right)}$ and splitting ratios $\Theta^{\left(t\right)}$ through the game-theory-based iterative method in the lower layer.
- Assess the impact on data rate $r_{i}$, energy harvesting $E_{i}$, and transmit power $P_{i}$, for all links *i*.
- Reward the action at time *t* as $U_{i}\left(P^{\left(t\right)},\theta_{i}^{\left(t\right)},F^{\left(t\right)}\right),\forall i$, based on the impact assessed.
- Train DQN with the system utility $U^{\left(t\right)}$ obtained.

After the training or learning period, say *T*, the trained DQN from Algorithm 3 would be used to observe the following state $s^{\left(t\right)}=\left\{L^{\left(t\right)},F^{\left(t\right)}\right\},t>T$, evaluate utility $U_{i}$ with the given parameters $\lambda_{i},\mu_{i}$, and $\nu_{i}$, and then take action $a^{\left(t\right)}$ to decide $P_{i}$, $\theta_{i}$, and $f_{i},\forall i$, for the system in the testing process.

### 4.5. Time Complexity

Next, we show the time complexity for each of these algorithms before revealing their performance differences in the next section. Specifically, let the number of episodes be M, and the number of time-steps per episode be N. Assuming that the Q-learning network in Algorithm 1 has *J* fully connected layers, the time complexity with regard to the number of (floating point) operations in this algorithm would be $O(\sum_{j=0}^{J-1}u_{j}u_{j+1})$ based on the analysis in [32], where $u_{j}$ denotes the unit number in the *j*th layer, and $u_{0}$ is the input state size. In each time-step of an episode, there may be other operations such as the random selection of an action in line 10 not involving the neural network, which could be ignored when compared with the former for the analysis. Thus, taking the nesting for loops (the outer is episode loop and the inner is time-step loop) into account, we have its worst-case time complexity as $O(MN\sum_{j=0}^{J-1}u_{j}u_{j+1})$.

Apart from training, DDPG also involves a normalization process whose time complexity could be denoted by T(s), where T(s) is the number of the variables in the state set. In addition, the actor and critic networks of DDPG in Algorithm 2 are assumed to have *J* and *K* fully connected layers, respectively. According to [32], the time complexity with respect to these networks in the training algorithm would be $O(\sum_{j=0}^{J-1}u_{actor,j}u_{actor,j+1}+\sum_{k=0}^{K-1}u_{critic,k}u_{critic,k+1})$, where $u_{actor,i}$ and $u_{critic,i}$ denote the unit number in the *i*th layer with respect to the actor network and the critic network, respectively. Then, by taking the nesting loops into account as well, we have the overall time complexity of this algorithm as $O(MN\left(\sum_{j=0}^{J-1}u_{actor,j}u_{actor,j+1}+\sum_{k=0}^{K-1}u_{critic,k}u_{critic,k+1}\right))$.

Finally, let the number of links be *n* and the number of iterations per link be K in addition to M and N given previously. As the two-layer hybrid training algorithm involves the lower-layer game-theory-based iterations, the overall time complexity of Algorithm 3 would be $O(MNnK\sum_{j=0}^{J-1}u_{Q,j}u_{Q,j+1})$, where $u_{Q,j}$ denotes the unit number in the *j*th layer with respect to the DQN neural network in this algorithm. Note that, although there are additional nK iterations for the lower layer, the input state size $u_{Q,0}$ is $\left|\left\{L^{\left(0\right)},F^{\left(0\right)}\right\}\right|$ that could be much smaller than $u_{0}=\left|\left\{L^{\left(0\right)},P^{\left(0\right)},\Theta^{\left(0\right)},F^{\left(0\right)}\right\}\right|$ in the single-layer Algorithm 1 with DQN, while $u_{Q,j}=u_{j},\;1\;\leq j\leq \;J$, is considered. In addition, it requires no normalization process and has the computational overhead on its neural network lower than that of $O(\sum_{j=0}^{J-1}u_{actor,j}u_{actor,j+1}+\sum_{k=0}^{K-1}u_{critic,k}u_{critic,k+1})$ on the two different types of neural networks in Algorithm 2.

## 5. Numerical Experiments

In this section, we conduct simulation experiments to evaluate the proposed two-layer approach and compare it with the single-layer approaches also introduced. To this end, we first present the simulation setup adopted and the parameters involved. Then, we show the performance differences between the two-layer hybrid MRA algorithm based on game theory and deep reinforcement learning, and the single-layer counterparts based on the conventional deep reinforcement learning models (DQN and DDPG).

### 5.1. Simulation Setup

With the network model and the channel model introduced in Section 2, we conduct MNs to be uniformly distributed in the simulated cellular network and let them move at a speed of *v* = 2 km/h on average with log-normal shadow fading as well as small-scale fading. In this environment, the cell radius is set to $\hat{r}$ and the distance between sites or BSs is considered to be 1.5 $\hat{r}$, in which MNs can experience a probability of line of sight, $P_{los}$, on the signals from BSs. For easy reference, the important parameters for the radio environment including those not shown above are summarized in Table 1.

Apart from the parameters for radio, the converge threshold ϱ is set to $10^{-5}$ for the two-layer algorithm, and the hyperparameters for the deep reinforcement learning models are tabulated in Table 2. For example, in the DQN for the single-layer approach, the state $s^{\left(t\right)}$ at time *t* is denoted by

$$\begin{array}{c} \begin{array}{c} \left\{X_{i}^{\left(t\right)},Y_{i}^{\left(t\right)},X_{j}^{\left(t\right)},Y_{j}^{\left(t\right)},P_{i}^{\left(t\right)},P_{j}^{\left(t\right)},\theta_{i}^{\left(t\right)},\theta_{j}^{\left(t\right)},f_{i}^{\left(t\right)},f_{j}^{\left(t\right)}\right\} \end{array} \end{array}$$

which corresponds to the size of state, 10, listed in this table. In addition, as introduced in Section 3.1, a ±1 dB offset representation is considered for PSF1, and the number of power levels is set here as 9 for PSF2 to construct their power sets $P^{1}$ and $P^{2}$, respectively. Furthermore, a ±0.05 offset representation, and a set of 11 values, $\left\{0,0.1,\cdots ,1\right\}$ with step size of 0.1, are also conducted as the power splitting ratio sets Θ for PSF1 and PSF2, respectively. Nevertheless, the size of action is 64 according to the binary decisions defined in (18), despite PSF1 or PSF2 in DQN. Apart from the above, for the two-layer approach, the DQN for the upper layer only considers the beamforming vectors F in addition to the locations L, which reduces the size of state to 6. Moreover, as it considers $\left\{-1,0,+1\right\}$ instead of ±1 for the actions, the size of action becomes 9. Despite these differences, the other hyperparameters of DQN are the same for both single- and two-layer approaches. Finally, the hyperparameters for DDPG are chosen to reflect its performance on average with a reasonable time complexity to execute, and a codebook F with 4, 8, 16, and 32 elements or vectors, respectively, to correspond to the different numbers of antennas in the radio environment is considered for all the algorithms involved.

Given that, we conduct 50 experiments with different seeds for all the algorithms under comparison. For each of these experiments, there are 400 training episodes or epochs in total. At the beginning of each episode, MNs are randomly located in the simulated network, which then move at speed *v* in 500 time slots per episode. Afterward, with the trained (P,Θ,F) from these algorithms, we conduct another 100 episodes with MNs randomly located at the beginning as well to obtain the averaged utility, data rate, energy harvesting, and power consumption to validate the parameters obtained with the different algorithms. Specifically, each 100 testing episodes of an experiment produce a mean value, and each averaged metric shown in the following figures denotes the average of these mean values from the 50 experiments. Note that, since DDPG is trained with normalized variables as shown in (32), in the testing process, we also have to preprocess these inputs.

### 5.2. Performance Comparison

**Figure 3:**
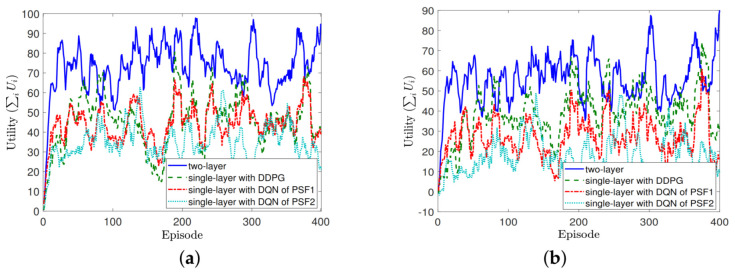
Utilities obtained during training periods upon (**a**) M=32, and (**b**) M=4.

**Figure 4:**
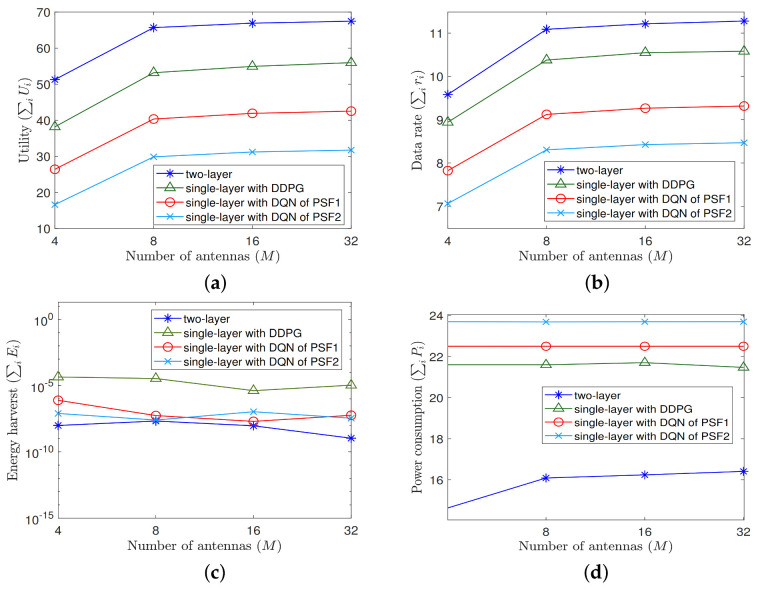
Impacts of varying the number of antennas (*M*) upon (**a**) utility, (**b**) data rate (bps), (**c**) energy harvesting (J), and (**d**) power consumption (W).

**Figure 5:**
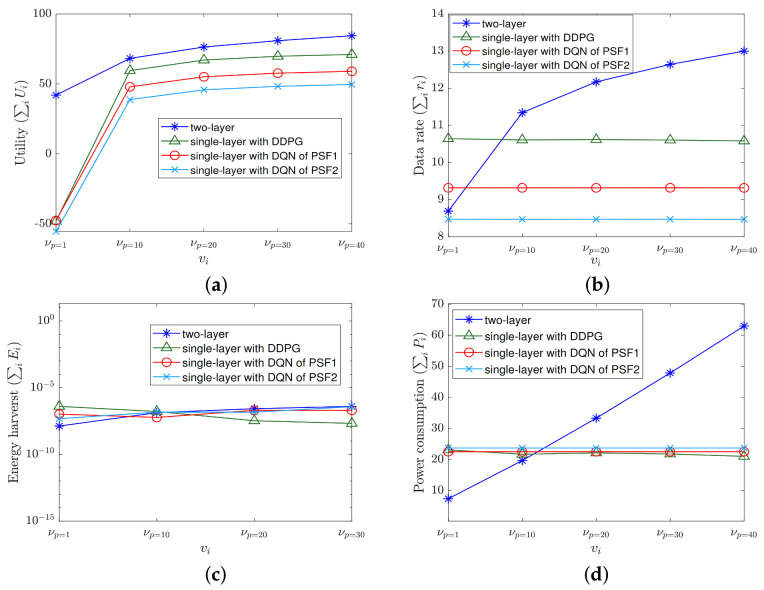
Impacts of the pricing strategy upon (**a**) utility, (**b**) data rate (bps), (**c**) energy harvesting (J), and (**d**) power consumption (W).

Given the environment, we compare the proposed two-layer MRA algorithm aided by game theory with the single-layer MRA algorithms based solely on DQN and DDPG also introduced. To see their performance differences, we conduct two sets of experiments from different aspects; the first focuses on the number of antennas, *M*, and the second on the power cost $\nu_{i}$. Given that, in Figure 3, Figure 4 and Figure 5 to be shown for the comparison results, the legends of “two-layer”, “single-layer with DDPG”, “single-layer with DQN of PSF1”, and “single-layer with DQN of PSF2” exhibited therein represent the two-layer MRA algorithm, the single-layer DDPG-based MRA algorithm, the single-layer DQN-based MRA algorithm with PSF1, and the single-layer DQN-based MRA algorithm with PSF2 introduced in this work, respectively.

#### 5.2.1. Impacts of Antennas

In the first experiment set, four numbers of transmit antennas, $M\in \left\{4,8,16,32\right\}$, in BS are examined while fixing $\lambda_{i}$ = 10, $\mu_{i}$ = 1, and $\nu_{i}$ = 1, ∀i. Due to similar trends to be given, in Figure 3, we exemplify the utilities obtained during the training periods in two experiment instances with the highest and the lowest number of transmit antennas, 32 and 4, respectively. It can be seen easily from the two sub-figures that the utility that resulted from the two-layer MRA algorithm is higher than those from the single-layer counterparts during the training periods, despite the number of antennas, on average. In addition, it can also be observed that, with the continuous action space, DDPG could outperform DQN in general, despite the power state formulations (PSF1 and PSF2) of the latter. Finally, we can see that, with a ±1 dB offset representation, PSF1 of DQN would result in a greater number of states on the transmit power than PSF2 equipped with a limited number of quantized levels, which could eventually lead to a better performance on the utility in the long term.

Next, we show the performance differences among the averaged metrics on utility, data rate, energy harvesting, and power consumption obtained by the testing process on (P,Θ,F) resulting from these algorithms. As shown in Figure 4, the two-layer MRA algorithm outperforms the single-layer counterparts on all the performance metrics except the energy harvesting, despite the number of antennas, *M*. In particular, in terms of the averaged utilities resulting from all different *M*, the two-layer MRA algorithm can achieve up to 2.3 times higher value than the single-layer DQN of the PSF2 algorithm. Despite the utility, as the resulting energy harvesting has relatively smaller values to impact the overall utility, a lower (resp. higher) value of this metric represented in the log scale is still possible and its impact would be compensated by a higher (resp. lower) value of power consumption, data rate, or both, which eventually leads to the overall utility to increase as *M* increases. For example, the highest utilities which are obtained by the two-layer MRA algorithm (as shown in Figure 4a) are mainly contributed by the highest data rates (as shown in Figure 4b) and the lowest power consumption (as shown in Figure 4d), which are all resulting from the two-layer algorithm, despite the energy harvesting of this algorithm to be slightly fluctuated as *M* increases and lower than that from the single-layer counterparts (as shown in Figure 4c).

In addition, as no previous works exactly consider the same system formulations and metrics presented here, it is hard to directly compare this work with the others such as [27,51] which consider only P, F, or both, for their data transmissions without the capability of energy harvesting. However, even without the capability, we could still consider the DRL algorithm in [51] with only P to see the possible performance differences between ours and the conventional approaches. Specifically, with M=32, the comparison results are summarized in Table 3. As shown readily, without the power split for energy harvesting, the DRL algorithm can obtain the highest data rate as an upper bound here, as expected. In comparison, the two-layer algorithm can achieve almost the same data rate while harvesting the energy with the lowest power consumption. Similarly, the single-layer algorithms can enjoy the energy harvesting with similar power consumption, but they may obtain lower data rates when splitting their powers to harvest energy and send data simultaneously.

#### 5.2.2. Impacts of Pricing Strategy

From the utility function defined by (19), we can see that the unit power cost $\nu_{i}$ actually plays a crucial role in the non-cooperative game model, and would have a strong impact on the performance of joint optimization and the Nash equilibrium. Thus, in the final set of experiments, we propose a simple pricing strategy for the base station to determine $\nu_{i}$ on the basis of social utility maximization and to control the transmit power of link so that its value can be located within the feasible range $\left[P_{min},P_{max}\right]$ for the high performance of this algorithm to be realized by the social utility maximization.

Specifically, let the desired transmit power be $P_{i}^{d}$, and, according to the fixed point formulation in (44), we have

(49) $$P_{i}^{d}=\frac{\lambda_{i}}{\nu_{i}-\frac{\mu_{i}{\left|h_{i,i}f_{i}\right|}^{2}}{{\hat{R}}_{i}+P_{i}^{d}{\left|h_{i,i}f_{i}\right|}^{2}}}-\frac{{\hat{R}}_{i}}{\left(1-\theta_{i}\right){\left|h_{i,i}f_{i}\right|}^{2}}$$

Given that, the desired power cost $\nu_{P_{i}^{d}}$ can be obtained by

(50) $$\nu_{P_{i}^{d}}=\frac{\mu_{i}{\left|h_{i,i}f_{i}\right|}^{2}}{{\hat{R}}_{i}+P_{i}^{d}{\left|h_{i,i}f_{i}\right|}^{2}}+\frac{\lambda_{i}\left(1-\theta_{i}\right){\left|h_{i,i}f_{i}\right|}^{2}}{P_{i}^{d}\left(1-\theta_{i}\right)+{\hat{R}}_{i}}$$

Accordingly, the two-layer hybrid MRA algorithm is slightly modified to dynamically adjust $\nu_{i}$ instead of using a fixed $\nu_{i},\forall i$, as an input of the algorithm. To be more specific, the sketch of this modification is given in Algorithm 4, wherein the modified three statements showing their calculations (50), (47) and (48), respectively, are highlighted with bold italic font, in addition to the fact that the input does not include $\nu_{i}$ now. For the comparison, the pricing strategy is also applied to Algorithms 1 and 2 by replacing the input $\nu_{i}$ with $\nu_{P_{i}^{d}}$ dynamically adjusted by using (50) as well after observing the next state $S^{\prime }$ carried out in the corresponding steps in these algorithms.
**Algorithm 4** The two-layer hybrid MRA training algorithm with the pricing strategy.(Input)$\lambda_{i},\mu_{i},\forall i$, ⋯;⋯**for** episode = 1 to M **do**    **for** time t=1 to N **do**      ⋯      Observe next state $S^{\prime }$;      **Obtain** $\nu_{P_{i}^{d}},\forall i$, **by using (50)**      **for** each link *i* **do**         **for** iteration k=1 to K **do**           **Update** $P_{i}\left[k\right]$**by using (47) with**$\nu_{i}=\nu_{P_{i}^{d}},\forall i$;           **Update** $\theta_{i}\left[k\right]$**by using (48) with**$\nu_{i}=\nu_{P_{i}^{d}},\forall i$;           ⋯         **end for**      **end for**      ⋯    **end for****end for**

Here, following the same setting $P_{min}$ = 1 W and $P_{max}$ = 40 W, we sample the feasible range at $\left\{1\;W,10\;W,20\;W,30\;W,40\;W\right\}$ as $P_{i}^{d}$ to obtain $\nu_{P_{i}^{d}}$ with (50) while fixing λ=10, μ=1, and M=32, and conduct these algorithms to output the performance metrics averaged to be compared. The results are now summarized in Figure 5, showing that the two-layer algorithm outperforms the others in terms of the utility. In particular, although it may have lower data rates when $\nu_{{}_{P=1}}$ (denoting $\nu_{P_{i}^{d}}$ obtained by $P_{i}^{d}=$ 1 W), and higher power consumption when $\nu_{{}_{P>10}}$ (denoting $\nu_{P_{i}^{d}}$ with $P_{i}^{d}>10$ W), the increasing trend of these resulting metrics would still lead to a utility higher than the others and the resulting utility would increase as $\nu_{P_{i}^{d}}$ increases. Similarly, as the energy harvesting has relatively smaller values to impact the system as noted before, its small fluctuations from the different algorithms do not alter the increasing trend of utility in the final experiment set as well.

## 6. Conclusions

In this work, we sought to maximize the utility that can make an optimal trade-off among data rate and energy harvesting while balancing the cost of power consumption in multi-access wireless networks with base stations having multi-antennas. Given the capability of selecting beamforming vectors from a finite set, adjusting transmit powers, and deciding power splitting ratios for energy harvesting, the wireless networks developed toward the future generation (beyond 5G or B5G) are expected to achieve the extreme performance requirements that can only be satisfied by an optimal solution to be possibly found through an exhaustive search.

To meet the expectation, we have shown in this work how to design DRL-based approaches operated in a single layer to jointly solve for power control, beamforming selection, and power splitting decision, and approach the optimal trade-off among the performance metrics without an exhaustive search in the action space that resulted. Furthermore, we have shown how to incorporate a data-driven DRL-based technique and a model-driven game-theory-based algorithm to form a two-layer iterative approach to resolve the NP-hard MRA problem in the wireless networks. Specifically, we have shown that, by taking benefits from both data-driven and model-driven methods, the proposed two-layer MRA algorithm can outperform the single-layer counterparts which rely only on the data-driven DRL-based algorithms. Here, the single-layer algorithms could represent the conventional DRL methods extended to have the energy harvesting capability. As shown readily in the experiments, the conventional DRL method and the single-layer algorithms would not provide a good performance trade-off on the metrics considered. That is, the overall utilities reflecting the trade-off from the single-layer algorithms have been shown to be lower than that from the two-layer approach. In contrast, by collaborating between DRL and game theory, the two-layer approach has been shown to achieve better trade-off among the data rate and the energy harvesting while balancing the cost of power consumption, reflecting on the higher utilities obtained. Specifically, in the simulation experiments, we have exemplified the performance differences of these algorithms in terms of data rate, energy harvesting, and power consumption, verified the feasibility of the three parameters in the utility function, and examined the pricing strategy proposed that can dynamically adjust the transmit power of the link to locate its value within the feasible range for the high performance of the two-layer algorithm to be obtained by the social utility maximization.

From the viewpoint of social utility maximization, our pricing strategy had been shown to give this system the leverage to select beamforming vectors, transmit powers, and power split ratios by properly adjusting the power costs. Finally, inspired by the related works on multi-agent DRL, we would aim to develop further collaborating schemes that can reduce the overhead caused by different optimization methods even under the non-stationary environment brought by a multi-agent setting, as our future work.

## Figures and Tables

**Table 1:** Important radio environment parameters.

Parameter	Value
Maximum transmit power ($P_{max}$)	40 W (46 dBm)
Minimum transmit power ($P_{min}$)	1 W (30 dBm)
Probability of light of sight ($P_{los}$)	0.7
Cell radius ($\hat{r}$)	150 m
Distance between sites (BSs)	225 m
Antenna gain	3 dBi
Mobile node (MN) antenna gain	0 dBi
Number of multipaths	4
MN movement speed on average (*v*)	2 km/h
Number of transmit antennas of BS	$\left\{4,8,16,32\right\}$
Downlink frequency band	28 GHz

**Table 2:** Reinforcement learning parameters.

Parameter	Value
*DQN*:	
Discount factor (ζ)	0.995
Learning rate (α)	0.01
Initial exploration rate (ϵ)	1.0
Minimum exploration rate ($\epsilon_{min}$)	0.1
Exploration decay rate (*d*)	0.9995
Size of state (|s|)	10
Size of action (|a|)	64
Replay buffer size (|D|)	2000
Batch size (η)	256
*DDPG*:	
Actor learning rate ($\alpha_{a}$)	0.001
Critic learning rate ($\alpha_{c}$)	0.002
Replay buffer size (|D|)	10000
Exploration decay rate (*d*)	0.9995
Batch size (η)	32
Scale factors ($\varsigma_{1}$, $\varsigma_{2}$, $\varsigma_{3}$)	1
Discount factor (ζ)	0.9
Soft update parameter (τ)	0.01
*DQN for two-layer*:	
Size of state (|s|)	6
Size of action (|a|)	9
The same parameters for the single-layer DQN	

**Table 3:** Performance comparison with M=32.

Method	Data Rate	Energy Harvesting	Power Consumption
DRL	11.32910	0	22.51510
two-layer	11.26969	$8.164853\times 10^{-9}$	16.40005
single-layer with DDPG	10.58339	$1.062941\times 10^{-5}$	21.34165
single-layer with DQN of PSF1	9.31607	$5.809001\times 10^{-8}$	22.50100
single-layer with DQN of PSF2	8.46842	$3.477011\times 10^{-8}$	23.69319

## Data Availability

Not applicable.

## Appendix A

**Table A1:** Important symbols for the proposed approaches in this work.

Name	Description	Name	Description
P,Θ,F	sets of transmit powers, splitting ratios, and beamforming vectors, respectively	$P_{i},\theta_{i},f_{i}$	transmit power, splitting ratio, and beamforming vector for link *i*, respectively
L	set of locations	$\hat{L}$	loss function
$\tilde{S}$	a finite set of states, $\left\{s_{1},s_{2},\cdots ,s_{m}\right\}$	$s^{\left(t\right)}$	state at time *t*, denoted by $\left\{L^{\left(t\right)}\right.$,$P^{\left(t\right)}$, $\Theta^{\left(t\right)}$, $\left.F^{\left(t\right)}\right\}$
$\tilde{A}$	a finite set of actions, $\left\{a_{1},a_{2},\cdots ,a_{n}\right\}$	$\hat{A}$	a set of binary variables, where ${\hat{A}}_{P}$, ${\hat{A}}_{\Theta }$, and ${\hat{A}}_{F}$ correspond to those for P, Θ, and F, respectively.
$\tilde{R}$	a finite set of rewards, where $\tilde{R}\left(s,a,s^{\prime }\right)$ is the function to provide reward r at state $s\in \tilde{S}$, action $a\in \tilde{A}$, and next state $s^{\prime }$	$\tilde{P}$	a finite set of transition probabilities, where ${\tilde{P}}_{ss^{\prime }\left(a\right)}=p\left(s^{\prime }|s,a\right)$ is the transition probability at state *s* taking action *a* to migrate to state $s^{\prime }$
$\pi^{*}\left(s\right)$	optimal policy at state *s*	$V^{\pi }\left(s\right)$	value function for the expected value to be obtained by policy π from state s∈S
$V^{*}\left(s\right)$	optimal action at state *s*	$Q^{\pi }\left(s,a\right)$	action–value function representing the expected reward starting from state *s* and taking action *a* from policy π
$Q^{\pi^{*}}$	optimal policy for the (optimal) action–value function $Q^{*}\left(s,a\right)=\max_{\pi }Q^{\pi }\left(s,a\right)$	$Q(s_{t},a_{t})$	action–value (Q) function at time *t*
$\hat{Q}(s_{t},a_{t},$ $\omega^{\prime })$	approximated action–value (Q) function with the weight of DNN, $\omega^{\prime }$, at time *t*	F	beamforming codebook
$P_{i}^{1}$	a set of transmit powers for link *i* in PSF1	$P^{2}$	a set of transmit powers for all links in PSF2
$U_{i}(P^{\left(t\right)},$ $\theta_{i}^{\left(t\right)},F^{\left(t\right)})$	reward for link *i* at time *t*, including data rate $r_{i}\left(P^{\left(t\right)},\theta_{i}^{\left(t\right)},F^{\left(t\right)}\right)$ and energy harvest $E_{i}(P^{\left(t\right)},\theta_{i}^{{}^{\left(t\right)}}$ $,F^{\left(t\right)})$	$(s^{\left(t\right)},a^{\left(t\right)},$ $r^{\left(t\right)},s^{\prime })$	transition at time *t*, where $r^{\left(t\right)}=U^{\left(t\right)}$ that is the system utility at this time step
α, $\alpha_{a}$, $\alpha_{c}$	learning rate, the (learning) rate specific to actor network, and the (learning) rate specific to critic network	$\epsilon ,\epsilon_{min}$	exploration rate (probability) and its minimum requirement
ζ	discount factor	τ	soft update parameter
*d*	exploration decay rate	*D*	replay buffer
η	batch size	ϱ	converge threshold for the fixed point iteration
$Q_{a}\left(s;\omega_{a}\right)$, $Q_{a^{\prime }}\left(s;\omega_{a^{\prime }}\right)$	output of actor network (online and target, respectively)	$Q(s,a;\omega_{c})$, $\bar{Q}\left(s,a;\omega_{c^{\prime }}\right)$	output of critic network (online and target, respectively)
$\alpha_{i},\mu_{i},\nu_{i}$	parameters for data rate, energy harvesting, and power consumption, respectively, for link *i*	$\varsigma_{1}$, $\varsigma_{2}$, $\varsigma_{3}$	scale factors for normalization of DDPG at time *t*
$a^{{\left(t\right)}^{*}}$	deterministic action of DDPG at time *t*, wherein $A_{P}^{{\left(t\right)}^{*}}$, $A_{\Theta }^{{\left(t\right)}^{*}}$, and $A_{F}^{{\left(t\right)}^{*}}$ correspond to those for transmit power, split ratio and beamforming vector	${\tilde{P}}_{i}^{\left(t\right)},{\tilde{\theta }}_{i}^{\left(t\right)}$, ${\tilde{f}}_{i}^{\left(t\right)}$	variables for normalization of DDPG at time *t*
$R_{i}$	total received power at link *i* for the fixed point iteration	${\hat{R}}_{i}$	auxiliary variable at link *i* for the fixed point iteration
$P_{i}^{d}$	desired transmit power at link *i* for the pricing strategy	$\nu_{P_{i}^{d}}$	desired power cost at link *i* for the pricing strategy

## References

[1] Zhang K., Mao Y., Leng S., Zhao Q., Li L., Peng X., Pan L., Maharjan S., Zhang Y. (2016). Energy-Efficient Offloading for Mobile Edge Computing in 5G Heterogeneous Networks. IEEE Access.

[2] Hewa T., Braeken A., Ylianttila M., Liyanage M. Multi-Access Edge Computing and Blockchain-based Secure Telehealth System Connected with 5G and IoT. Proceedings of the GLOBECOM 2020—2020 IEEE Global Communications Conference.

[3] Chen F., Wang A., Zhang Y., Ni Z., Hua J. (2021). Energy Efficient SWIPT Based Mobile Edge Computing Framework for WSN-Assisted IoT. Sensors.

[4] Chae S.H., Jeong C., Lim S.H. (2018). Simultaneous Wireless Information and Power Transfer for Internet of Things Sensor Networks. IEEE Internet Things J..

[5] Masood Z.A., Choi Y. (2021). Energy-efficient optimal power allocation for swipt based iot-enabled smart meter. Sensors.

[6] Liu J.-S., Lin C.-H.R., Tsai J. (2017). Delay and energy trade-off in energy harvesting multi-hop wireless networks with inter-session network coding and successive interference cancellation. IEEE Access.

[7] Tran T.-N., Voznak M. (2021). Switchable Coupled Relays Aid Massive Non-Orthogonal Multiple Access Networks with Transmit Antenna Selection and Energy Harvesting. Sensors.

[8] Luo Z.-Q., Zhang S. (2008). Dynamic Spectrum Management: Complexity and Duality. IEEE J. Sel. Top. Signal Process..

[9] Boccardi F., Heath R.W., Lozano A., Marzetta T.L., Popovski P. (2014). Five disruptive technology directions for 5G. IEEE Commun. Mag..

[10] Li Y., Luo J., Xu W., Vucic N., Pateromichelakis E., Caire G. A Joint Scheduling and Resource Allocation Scheme for Millimeter Wave Heterogeneous Networks. Proceedings of the 2017 IEEE Wireless Communications and Networking Conference (WCNC).

[11] Yang Z., Xu W., Xu H., Shi J., Chen M. User Association, Resource Allocation and Power Control in Load-Coupled Heterogeneous Networks. Proceedings of the 2016 IEEE Globecom Workshops (GC Wkshps).

[12] Saeed A., Katranaras E., Dianati M., Imran M.A. (2016). Dynamic femtocell resource allocation for managing inter-tier interference in downlink of heterogeneous networks. IET Commun..

[13] Coskun C.C., Davaslioglu K., Ayanoglu E. (2017). Three-Stage Resource Allocation Algorithm for Energy-Efficient Heterogeneous Networks. IEEE Trans. Veh. Technol..

[14] Liu R., Sheng M., Wu W. (2018). Energy-Efficient Resource Allocation for Heterogeneous Wireless Network With Multi-Homed User Equipments. IEEE Access.

[15] Le N.-T., Tran L.-N., Vu Q.-D., Jayalath D. (2019). Energy-Efficient Resource Allocation for OFDMA Heterogeneous Networks. IEEE Trans. Commun..

[16] Zhang Y., Wang Y., Zhang W. Energy efficient resource allocation for heterogeneous cloud radio access networks with user cooperation and QoS guarantees. Proceedings of the 2016 IEEE Wireless Communications and Networking Conference.

[17] Zou S., Liu N., Pan Z., You X. Joint Power and Resource Allocation for Non-Uniform Topologies in Heterogeneous Networks. Proceedings of the 2016 IEEE 83rd Vehicular Technology Conference (VTC Spring).

[18] Zhang H., Du J., Cheng J., Long K., Leung V.C.M. (2017). Incomplete CSI Based Resource Optimization in SWIPT Enabled Heterogeneous Networks: A Non-Cooperative Game Theoretic Approach. IEEE Trans. Wirel. Commun..

[19] Chen X., Zhao Z., Zhang H. (2012). Stochastic Power Adaptation with Multiagent Reinforcement Learning for Cognitive Wireless Mesh Networks. IEEE Trans. Mob. Comput..

[20] Xu C., Sheng M., Yang C., Wang X., Wang L. (2013). Pricing-Based Multiresource Allocation in OFDMA Cognitive Radio Networks: An Energy Efficiency Perspective. IEEE Trans. Veh. Technol..

[21] Jiang Y., Lu N., Chen Y., Zheng F., Bennis M., Gao X., You X. (2017). Energy-Efficient Noncooperative Power Control in Small-Cell Networks. IEEE Trans. Veh. Technol..

[22] Zhang H., Song L., Han Z. (2016). Radio Resource Allocation for Device-to-Device Underlay Communication Using Hypergraph Theory. IEEE Trans. Wirel. Commun..

[23] Zhang R., Cheng X., Yang L., Jiao B. Interference-aware graph based resource sharing for device-to-device communications underlaying cellular networks. Proceedings of the 2013 IEEE Wireless Communications and Networking Conference (WCNC).

[24] Feng D., Lu L., Yuan-Wu Y., Li G.Y., Feng G., Li S. (2013). Device-to-device communications underlaying cellular networks. IEEE Trans. Commun..

[25] Jiang Y., Liu Q., Zheng F., Gao X., You X. (2016). Energy-Efficient Joint Resource Allocation and Power Control for D2D Communications. IEEE Trans. Veh. Technol..

[26] Mnih V., Kavukcuoglu K., Silver D., Rusu A.A., Veness J., Bellemare M.G., Graves A., Riedmiller M., Fidjeland A.K., Ostrovski G. (2015). Human-level control through deep reinforcement learning. Nature.

[27] Meng F., Chen P., Wu L., Cheng J. (2020). Power Allocation in Multi-User Cellular Networks: Deep Reinforcement Learning Approaches. IEEE Trans. Wirel. Commun..

[28] Nguyen K.K., Duong T.Q., Vien N.A., Le-Khac N.-A., Nguyen M.-N. (2019). Non-Cooperative Energy Efficient Power Allocation Game in D2D Communication: A Multi-Agent Deep Reinforcement Learning Approach. IEEE Access.

[29] Zhang Y., Kang C., Ma T., Teng Y., Guo D. Power Allocation in Multi-Cell Networks Using Deep Reinforcement Learning. Proceedings of the 2018 IEEE 88th Vehicular Technology Conference (VTC-Fall).

[30] Choi J. (2014). Massive MIMO With Joint Power Control. IEEE Wirel. Commun. Lett..

[31] Zhang Y., Kang C., Teng Y., Li S., Zheng W., Fang J. Deep Reinforcement Learning Framework for Joint Resource Allocation in Heterogeneous Networks. Proceedings of the 2019 IEEE 90th Vehicular Technology Conference (VTC2019-Fall).

[32] Qiu C., Hu Y., Chen Y., Zeng B. (2019). Deep Deterministic Policy Gradient (DDPG)-Based Energy Harvesting Wireless Communications. IEEE Internet Things J..

[33] 3GPP (2015). Evolved Universal Terrestrial Radio Access (E-UTRA): Physical Layer Procedures (3GPP).

[34] Kim R., Kim Y., Yu N.Y., Kim S.-J., Lim H. (2019). Online Learning-Based Downlink Transmission Coordination in Ultra-Dense Millimeter Wave Heterogeneous Networks. IEEE Trans. Wirel. Commun..

[35] Song Q., Wang X., Qiu T., Ning Z. (2017). An Interference Coordination-Based Distributed Resource Allocation Scheme in Heterogeneous Cellular Networks. IEEE Access.

[36] Trakas P., Adelantado F., Zorba N., Verikoukis C. A QoE-aware joint resource allocation and dynamic pricing algorithm for heterogeneous networks. Proceedings of the GLOBECOM 2017—2017 IEEE Global Communications Conference.

[37] Simsek M., Bennis M., Guvenc I. (2014). Learning Based Frequency- and Time-Domain Inter-Cell Interference Coordination in HetNets. IEEE Trans. Veh. Technol..

[38] Ghadimi E., Calabrese F.D., Peters G., Soldati P. A reinforcement learning approach to power control and rate adaptation in cellular networks. Proceedings of the 2017 IEEE International Conference on Communications (ICC).

[39] Calabrese F.D., Wang L., Ghadimi E., Peters G., Hanzo L., Soldati P. (2018). Learning Radio Resource Management in RANs: Framework, Opportunities, and Challenges. IEEE Commun. Mag..

[40] Sharma S., Darak S.J., Srivastava A. Energy saving in heterogeneous cellular network via transfer reinforcement learning based policy. Proceedings of the 2017 9th International Conference on Communication Systems and Networks (COMSNETS).

[41] Wei Y., Yu F.R., Song M., Han Z. (2017). User Scheduling and Resource Allocation in HetNets With Hybrid Energy Supply: An Actor-Critic Reinforcement Learning Approach. IEEE Trans. Wirel. Commun..

[42] Liang L., Feng G. (2011). A Game-Theoretic Framework for Interference Coordination in OFDMA Relay Networks. IEEE Trans. Veh. Technol..

[43] Lu Y., Xiong K., Fan P., Zhong Z., Ai B., Ben Letaief K. (2021). Worst-Case Energy Efficiency in Secure SWIPT Networks with Rate-Splitting ID and Power-Splitting EH Receivers. IEEE Trans. Wirel. Commun..

[44] Xu Y., Li G., Yang Y., Liu M., Gui G. (2019). Robust Resource Allocation and Power Splitting in SWIPT Enabled Heterogeneous Networks: A Robust Minimax Approach. IEEE Internet Things J..

[45] Zhang R., Xiong K., Lu Y., Gao B., Fan P., Ben Letaief K. (2021). Joint Coordinated Beamforming and Power Splitting Ratio Optimization in MU-MISO SWIPT-Enabled HetNets: A Multi-Agent DDQN-Based Approach. IEEE J. Sel. Areas Commun..

[46] Omidkar A., Khalili A., Nguyen H.H., Shafiei H. (2022). Reinforcement Learning Based Resource Allocation for Energy-Harvesting-Aided D2D Communications in IoT Networks. IEEE Internet Things J..

[47] Canese L., Cardarilli G., Di Nunzio L., Fazzolari R., Giardino D., Re M., Spanò S. (2021). Multi-Agent Reinforcement Learning: A Review of Challenges and Applications. Appl. Sci..

[48] Sutton R.S., Barto A.G. (2018). Reinforcement Learning: An Introduction.

[49] Goodfellow J., Pouget-Abadie J., Mirza M., Xu B., Warde-Farley D., Ozair S., Courville A., Bengio Y. Generative adversarial nets. Proceedings of the Advances in Neural Information Processing Systems 27: Annual Conference on Neural Information Processing Systems 2014.

[50] Perera T.D.P., Jayakody D.N.K., Sharma S.K., Chatzinotas S., Li J. (2017). Simultaneous Wireless Information and Power Transfer (SWIPT): Recent Advances and Future Challenges. IEEE Commun. Surv. Tutor..

[51] Mismar F.B., Evans B.L., Alkhateeb A. (2019). Deep Reinforcement Learning for 5G Networks: Joint Beamforming, Power Control, and Interference Coordination. IEEE Trans. Commun..

[52] Alkhateeb A., El Ayach O., Leus G., Heath R.W. (2014). Channel Estimation and Hybrid Precoding for Millimeter Wave Cellular Systems. IEEE J. Sel. Top. Signal Process..

[53] Heath R.W., Gonzalez-Prelcic N., Rangan S., Roh W., Sayeed A.M. (2016). An Overview of Signal Processing Techniques for Millimeter Wave MIMO Systems. IEEE J. Sel. Top. Signal Process..

[54] Schniter P., Sayeed A. Channel estimation and precoder design for millimeter-wave communications: The sparse way. Proceedings of the 2014 48th Asilomar Conference on Signals, Systems and Computers.

[55] Rappaport T., Gutierrez F., Ben-Dor E., Murdock J.N., Qiao Y., Tamir J.I. (2013). Broadband Millimeter-Wave Propagation Measurements and Models Using Adaptive-Beam Antennas for Outdoor Urban Cellular Communications. IEEE Trans. Antennas Propag..

[56] Rappaport T.S., Heath R.W., Daniels R.C., Murdock J.N. (2014). Millimeter Wave Wireless Communications.

[57] Lu X., Wang P., Niyato D., Kim D.I., Han Z. (2015). Wireless charging technologies: Fundamentals, standards, and network applications. IEEE Commun. Surv. Tutor..

[58] Ng D.W.K., Lo E.S., Schober R. (2016). Multiobjective Resource Allocation for Secure Communication in Cognitive Radio Networks With Wireless Information and Power Transfer. IEEE Trans. Veh. Technol..

[59] Chang Z., Gong J., Ristaniemi T., Niu Z. (2016). Energy-Efficient Resource Allocation and User Scheduling for Collaborative Mobile Clouds With Hybrid Receivers. IEEE Trans. Veh. Technol..

[60] Sen S., Santhapuri N., Choudhury R.R., Nelakuditi S. Successive interference cancellation: A back-of-the-envelope perspective. Proceedings of the 9th ACM SIGCOMM Workshop on Hot Topics in Networks.

[61] Bertsekas D.P. (1995). Dynamic Programming and Optimal Control.

[62] Li X., Fang J., Cheng W., Duan H., Chen Z., Li H. (2018). Intelligent Power Control for Spectrum Sharing in Cognitive Radios: A Deep Reinforcement Learning Approach. IEEE Access.

[63] Ioffe S., Szegedy C. Batch normalization: Accelerating deep network training by reducing internal covariate shift. Proceedings of the 32nd International Conference on Machine Learning.

[64] Sutton R.S., Barto A.G. (1998). Introduction to Reinforcement Learning.

[65] Alkhateeb A., Alex S., Varkey P., Li Y., Qu Q., Tujkovic D. (2018). Deep Learning Coordinated Beamforming for Highly-Mobile Millimeter Wave Systems. IEEE Access.

[66] Fudenberg D., Tirole J. (1991). Game Theory.

